# Supplementary material for: Ether phospholipids are required for mitochondrial reactive oxygen species homeostasis
Source: Nat Commun. 2023 Apr 17;14:2194. doi: 10.1038/s41467-023-37924-9 (PMC10110566; doi:10.1038/s41467-023-37924-9)
Supplement: Supplementary file 1 — Supplementary Information [file 41467_2023_37924_MOESM1_ESM.pdf]

# Ether phospholipids are required for mitochondrial reactive oxygen species homeostasis

## List of Supplementary Information

## Supplementary Figures

Supplementary Figure 1. Sensitivity of PDAC PDX lines to different mitochondrial inhibitors.

Supplementary Figure 2. Mitochondrial complex I inhibition in PDAC PDX cells grown in glucose-rich or -limited condition.

Supplementary Figure 3. Sensitivity comparation to complex I inhibition in PDX cells grown in galactose medium or hypoxic condition.

Supplementary Figure 4. Differential responses of PDAC PDX models to treatment with mitochondrial complex I inhibitor IACS-010759.

Supplementary Figure 5. Sensitivity of PDAC PDX tumors to treatment with mitochondrial complex I inhibitor.

Supplementary Figure 6. Comparation of mitochondrial quality and quantity in complex I inhibition-sensitive and -resistant lines.

Supplementary Figure 7. Mitochondrial ROS accumulation was happened earlier than IACS-010759 induced cell death in sensitive lines.

Supplementary Figure 8. Two pH-stable GFP probes for hydrogen peroxide monitor mitochondrial localized-ROS and cytosol localized-ROS.

Supplementary Figure 9. Mitochondrial ROS induction increases cell sensitivity to IACS-010759.

Supplementary Figure 10. Higher TCA cycle components in resistant group.

Supplementary Figure 11. MUFAs and PUFAs exhibit opposite contribution to cell survival and ROS homeostasis upon mitochondrial complex I inhibition.

Supplementary Figure 12. Complex I inhibition induces lipid peroxidation in cells sensitive to IACS-010759 treatment.

Supplementary Figure 13. A deep dissection of cell death mechanism induced by IACS-010759.

Supplementary Figure 14. Blocking de novo ether phospholipid synthesis by knocking out key ether lipids generation enzymes.

Supplementary Figure 15. Supplementation with C18:1-linked ether lipids (O-C16-18:1 PC) induce accumulation in mitochondria.

Supplementary Figure 16. Peroxisome activities comparation between sensitive and resistant lines.

Supplementary Figure 17. GNPAT-deleted tumors with IACS-010759 increased cell death and lipids peroxidation.

Supplementary Figure 18. MUFA-linked ether phosphatidylcholine promotes resistance to mitochondrial complex I inhibition.

Supplementary Figure 19. MUFA-linked ether phosphatidylcholine rescue cell death induced by mitochondrial complex I inhibitor phenformin.

Supplementary Figure 20. Blue native PAGE and In-gel activity protocols identified mitochondrial supercomplexes.

Supplementary Figure 21. MUFA-linked ether phosphatidylcholine promotes the formation of high-molecular-weight supercomplexes.

Supplementary Figure 22. Decrease to SCs assembly by knocking out UQCC3 sensitize to complex I inhibition.

## Supplementary Tables

Supplementary Table 1. IC50 for IACS-010759 in PDXs.

Supplementary Table 2. Main genetic mutation features in PDXs.

## Supplementary Data

Supplementary Data 1. Global polar metabolomics in PDX lines.

Supplementary Data 2. Whole cell lipidomics in PDX lines.

Supplementary Data 3. Lipidomics in purified mitochondria of PDX lines.

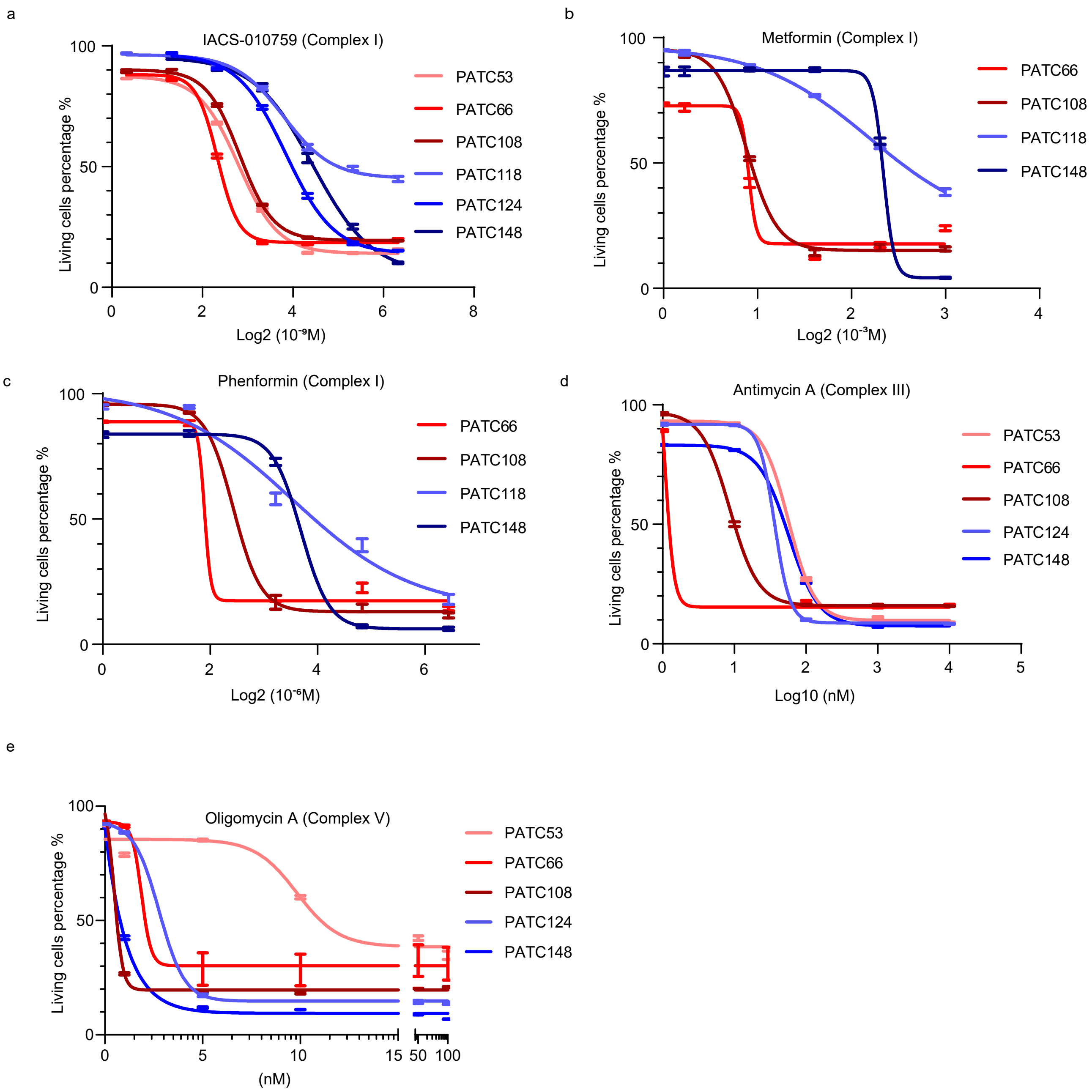
a
IACS-010759 (Complex I)
Living cells percentage %
Log2 (10⁻⁹M)
PATC53
PATC66
PATC108
PATC118
PATC124
PATC148
b
Metformin (Complex I)
Living cells percentage %
Log2 (10⁻³M)
PATC66
PATC108
PATC118
PATC148
c
Phenformin (Complex I)
Living cells percentage %
Log2 (10⁻⁶M)
PATC66
PATC108
PATC118
PATC148
d
Antimycin A (Complex III)
Living cells percentage %
Log10 (nM)
PATC53
PATC66
PATC108
PATC124
PATC148
e
Oligomycin A (Complex V)
Living cells percentage %
(nM)
PATC53
PATC66
PATC108
PATC124
PATC148

**Supplementary Figure 1. Sensitivity of PDAC PDX lines to different mitochondrial inhibitors.** Dose-response curves in representative PATC lines grown in 5.5 mM glucose and treated for 72 hours with complex I inhibitors IACS-010759 (a), metformin (b), phenformin (c), or complex III inhibitor antimycin A (d), or complex V inhibitor oligomycin A (e) at the indicated concentration. Cell death was detected by propidium iodide staining and flow cytometry Data represent mean ± S.D of 3 biologically independent replicates (a-e).

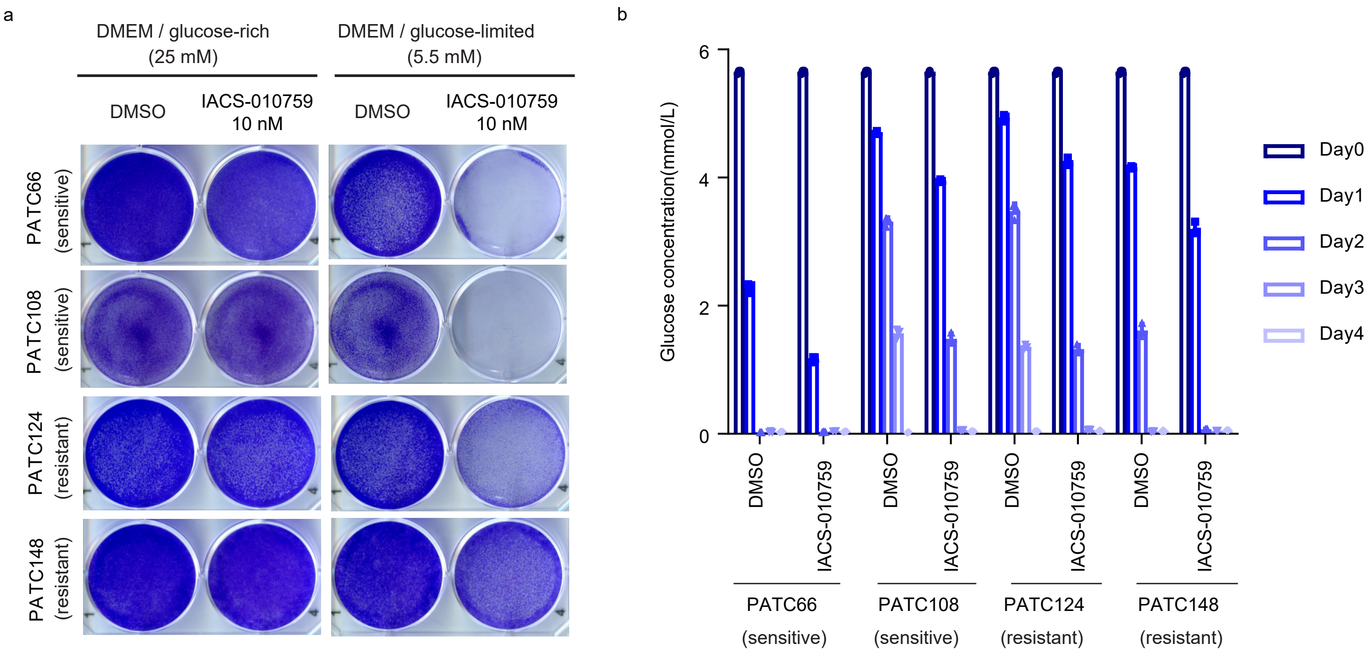
a
DMEM / glucose-rich (25 mM)
DMEM / glucose-limited (5.5 mM)
DMSO
IACS-010759 10 nM
PATC66 (sensitive)
PATC108 (sensitive)
PATC124 (resistant)
PATC148 (resistant)
b
Glucose concentration(mmol/L)
0
2
4
6
Day0
Day1
Day2
Day3
Day4
DMSO
IACS-010759
PATC66 (sensitive)
PATC108 (sensitive)
PATC124 (resistant)
PATC148 (resistant)

**Supplementary Figure 2. Mitochondrial complex I inhibition in PDAC PDX cells grown in glucose-rich or -limited condition.** a) Crystal violet staining of cells grown in the presence or absence of 10nM IACS-010759 in 25 mM or 5.5 mM glucose for 4 days. b) Medium glucose concentration was detected in sensitive lines (PATC66/108) and resistant lines (PATC124/148) with DMSO or 10 nM IACS-010759 treatment for 0, 1, 2, 3, 4-days. Data represent mean ± S.D of 3 biologically independent replicates.

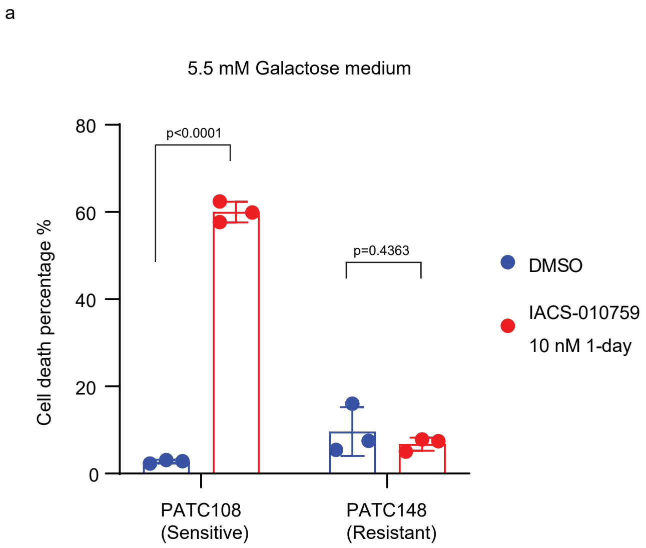
a
5.5 mM Galactose medium
Cell death percentage %
80
60
40
20
0
p<0.0001
p=0.4363
PATC108 (Sensitive)
PATC148 (Resistant)
DMSO
IACS-010759 10 nM 1-day

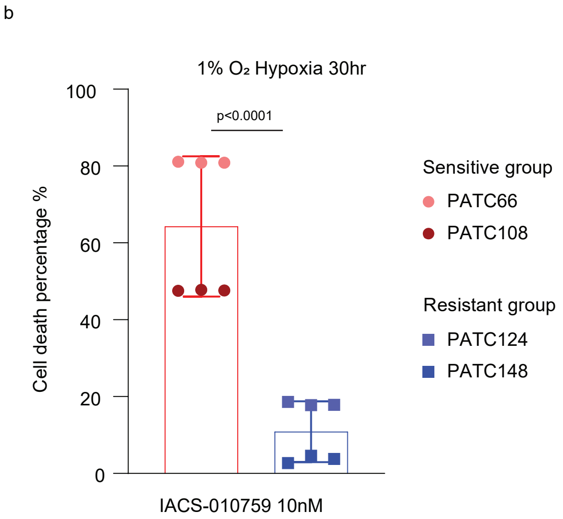
b
1% O₂ Hypoxia 30hr
Cell death percentage %
100
80
60
40
20
0
p<0.0001
IACS-010759 10nM
Sensitive group
PATC66
PATC108
Resistant group
PATC124
PATC148

**Supplementary Figure 3. Sensitivity comparation to complex I inhibition in PDX cells grown in galactose medium or hypoxic condition.** a) PATC108 (sensitive) and PATC148 (resistant) cells were treated with DMSO or 10 nM IACS-010759 for 1-day in 5.5 mM galactose medium. Cell viability were detected by PI stain. Data represent mean ± S.D of 3 biologically independent replicates. b) Cell viability assay assessing response of sensitive (PATC66/108) and resistant (PATC124/148) PDX lines to IACS-010759 (10 nM) for 30 hours under hypoxic conditions (1% $O_2$). 3 biologically independent replicates per cell line. Data represent mean ± S.D between sensitive group (2 cell lines) and resistant group (2 cell lines). Statistical analysis by two-tailed Students' unpaired t test with significance indicated (a, b). Source data are provided as a Source Data file.

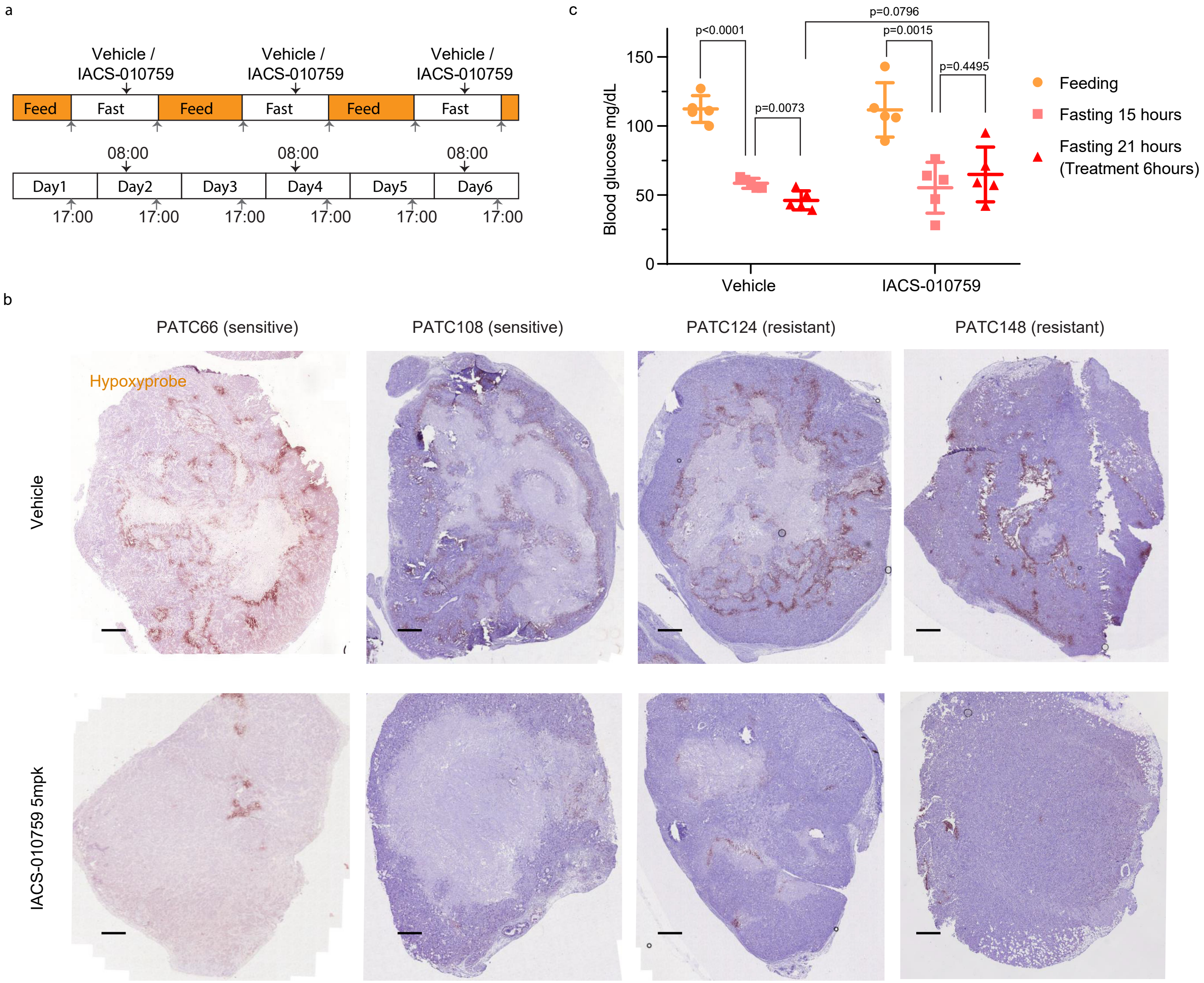
a
Vehicle / IACS-010759
Feed
Fast
08:00
Day1
Day2
Day3
Day4
Day5
Day6
17:00
b
PATC66 (sensitive)
PATC108 (sensitive)
PATC124 (resistant)
PATC148 (resistant)
Hypoxyprobe
Vehicle
IACS-010759 5mpk
c
Blood glucose mg/dL
p<0.0001
p=0.0073
p=0.0796
p=0.0015
p=0.4495
Vehicle
IACS-010759
Feeding
Fasting 15 hours
Fasting 21 hours (Treatment 6hours)

**Supplementary Figure 4. Differential responses of PDAC PDX models to treatment with mitochondrial complex I inhibitor IACS-010759.** a) Schematic representation of fasting/feeding protocols and timing of IACS-010759 administration in the PDX models. b) Representative IHC images showing hypoxia (Hypoxyprobe, pimonidazole) staining of PATCs xenograft tumors, from mice treated with vehicle or 5 mg/kg IACS-010759 at the $15^{th}$ day. (scale bar = 500 µm). c) Blood glucose level were monitored at the indicated times upon feeding or fasting, in mice treated with vehicle or 5mg/kg IACS-010759 once every other day. Data represent mean ± S.D of 5 biologically independent replicates. Statistical analysis by two-tailed Students' unpaired t test with significance indicated (c). Source data are provided as a Source Data file.

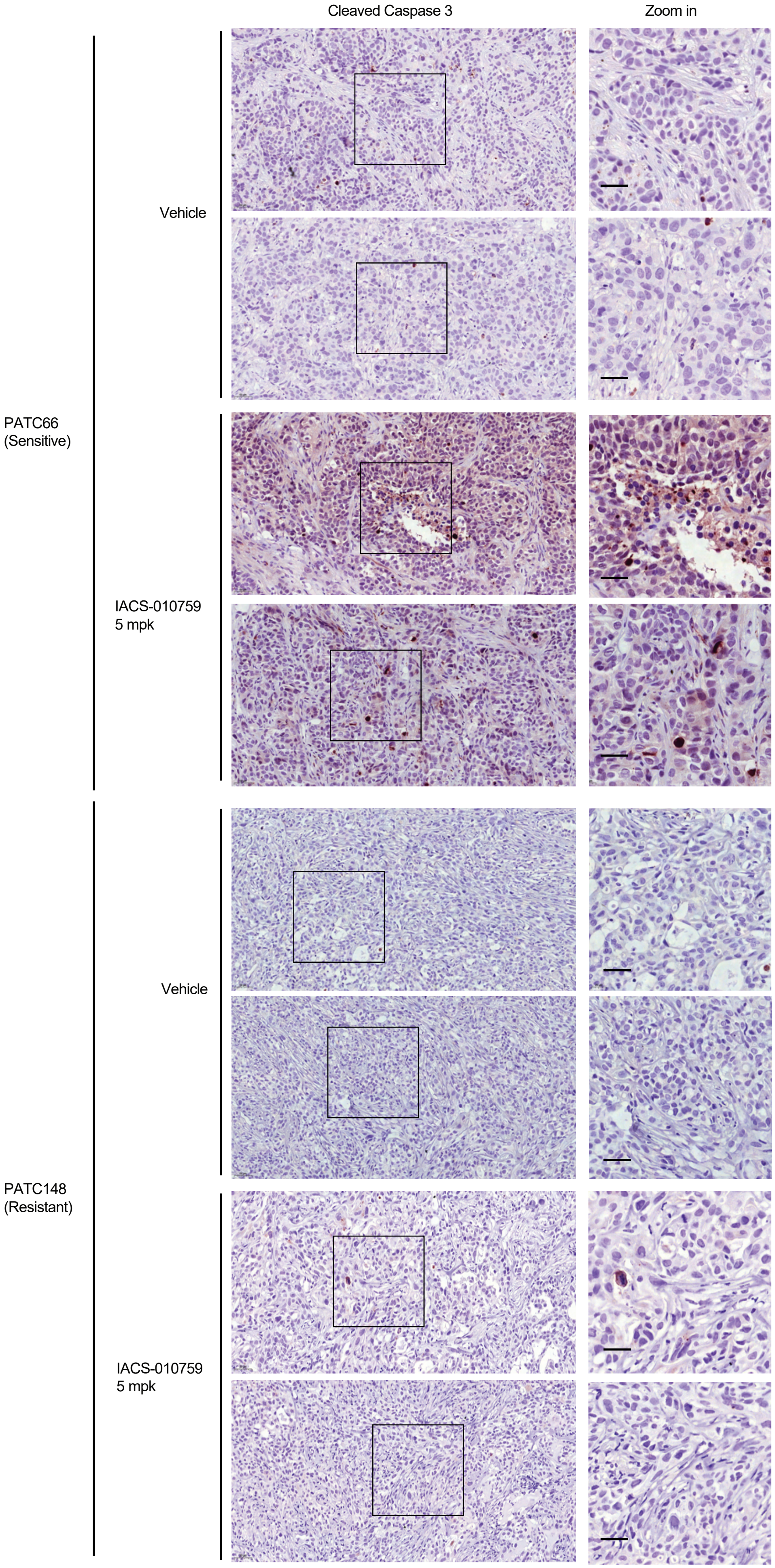

Cleaved Caspase 3
Zoom in
PATC66
(Sensitive)
Vehicle
IACS-010759
5 mpk
PATC148
(Resistant)
Vehicle
IACS-010759
5 mpk

**Supplementary Figure 5. Sensitivity of PDAC PDX tumors to treatment with mitochondrial complex I inhibitor.** Representative IHC for cleaved caspase 3 activity in PATC subcutaneous tumors treated with vehicle or 5mg/kg IACS-010759 on the 23$^{rd}$ (PATC66) and 26$^{th}$ (PATC148) days. For each treatment, 2 independent tumors were stained. Scale bar, 50 μm.

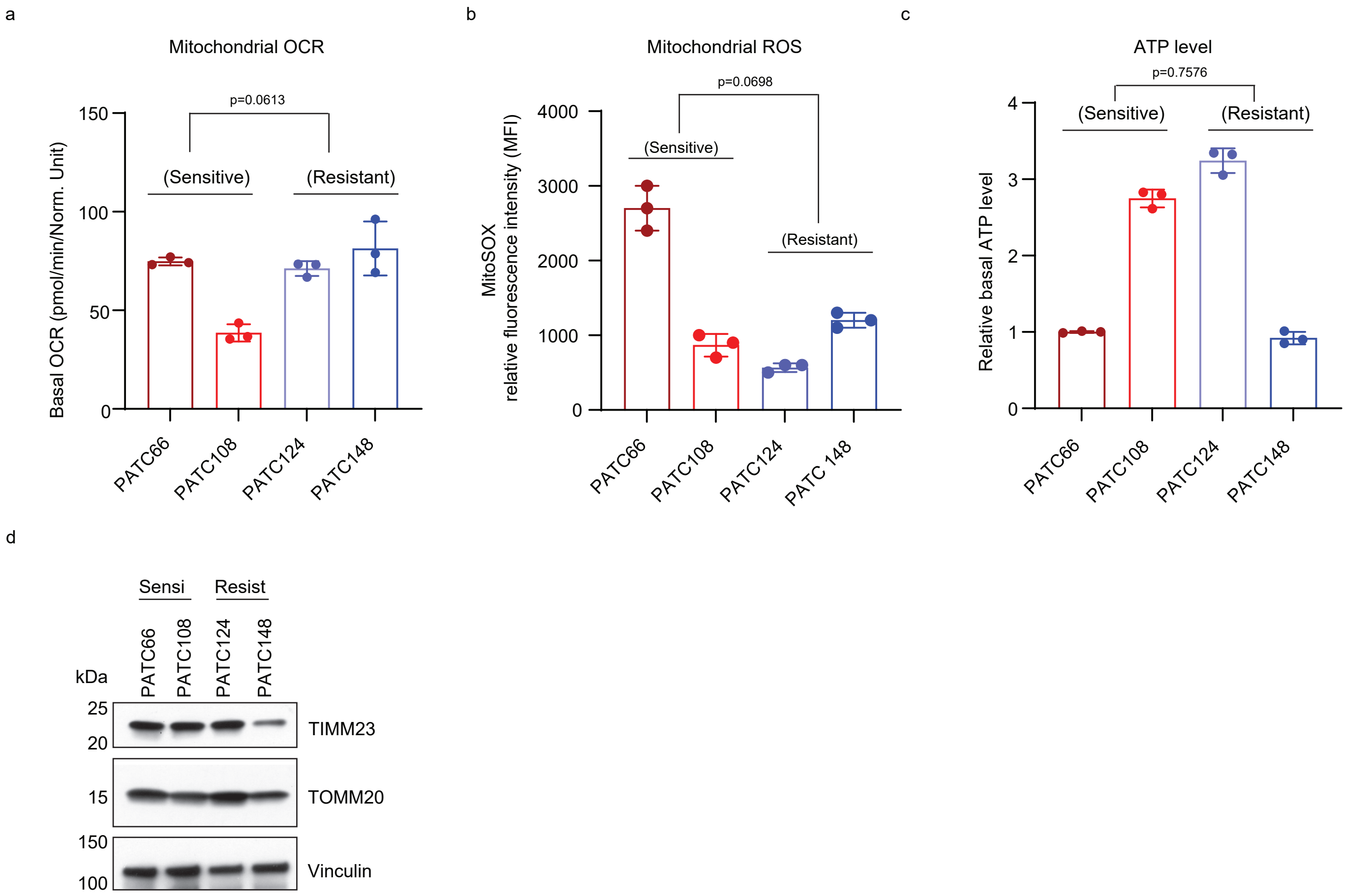
a
Mitochondrial OCR
p=0.0613
(Sensitive)
(Resistant)
Basal OCR (pmol/min/Norm. Unit)
150
100
50
0
PATC66
PATC108
PATC124
PATC148
b
Mitochondrial ROS
p=0.0698
(Sensitive)
(Resistant)
MitoSOX
relative fluorescence intensity (MFI)
4000
3000
2000
1000
0
PATC66
PATC108
PATC124
PATC 148
c
ATP level
p=0.7576
(Sensitive)
(Resistant)
Relative basal ATP level
4
3
2
1
0
PATC66
PATC108
PATC124
PATC148
d
Sensi
Resist
PATC66
PATC108
PATC124
PATC148
kDa
25
20
TIMM23
15
TOMM20
150
100
Vinculin

**Supplementary Figure 6. Comparation of mitochondrial quality and quantity in complex I inhibition-sensitive and -resistant lines.** Comparation of Basal oxygen consumption ratio (a), mitochondrial ROS by mito-SOX stain (b) and ATP level (c) in PATC66 / PATC108 / PATC124 / PATC148 lines. Data represent mean ± S.D of 3 biologically independent replicates. d) Western blotting showed mitochondrial mass (TIMM23, mitochondrial inner membrane protein; TOMM20, mitochondrial outer membrane protein) in sensitive (PATC66/108) and resistant (PATC124/148) cells. Statistical analysis by two-tailed Students' unpaired t test with significance indicated (a-c). Source data are provided as a Source Data file.

a

Gating strategy for flow cytometry (MitoSOX stain as an example)

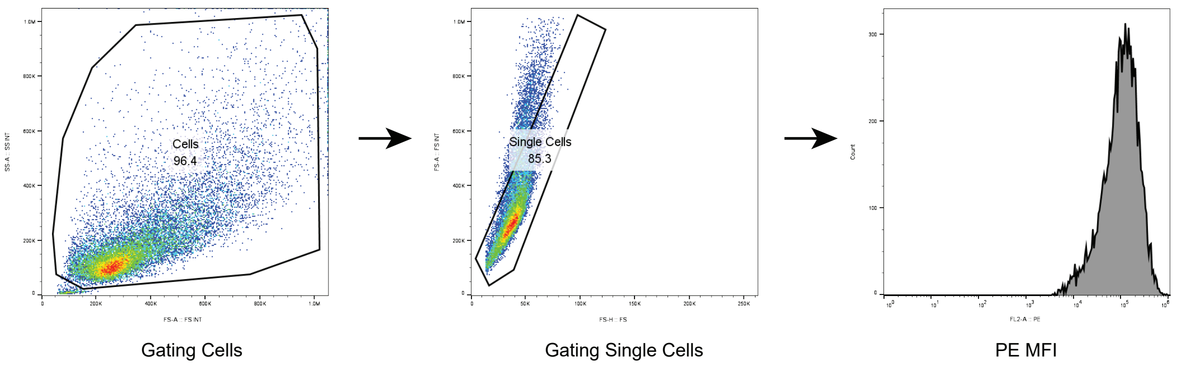


b

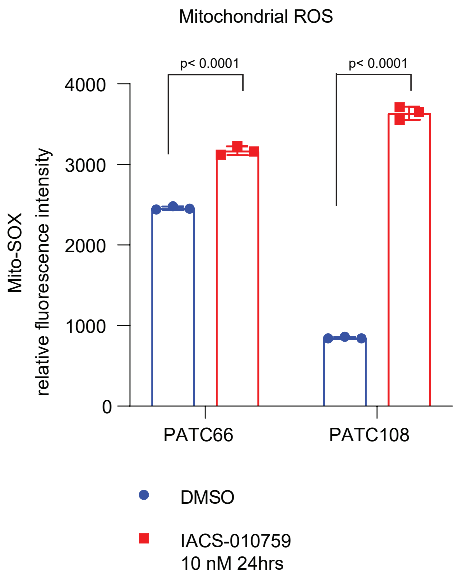


c

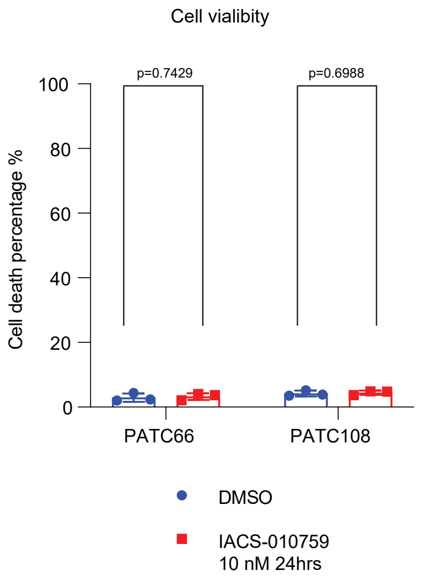

**Supplementary Figure 7. Mitochondrial ROS accumulation was happened earlier than IACS-010759 induced cell death in sensitive lines.** a) Flow cytometry gating strategy were used in MitoSOX data analysis. b-c) Sensitive cell lines PATC66 and 108 were treated with DMSO or 10nM IACS-010759 for 24hr. Mitochondrial ROS (b) and cell viability (c) were detected by mito-ROS and PI stain, separately. Data represent mean ± S.D of 3 biologically independent replicates. Statistical analysis by two-tailed Students' unpaired t test with significance indicated (b, c). Source data are provided as a Source Data file.

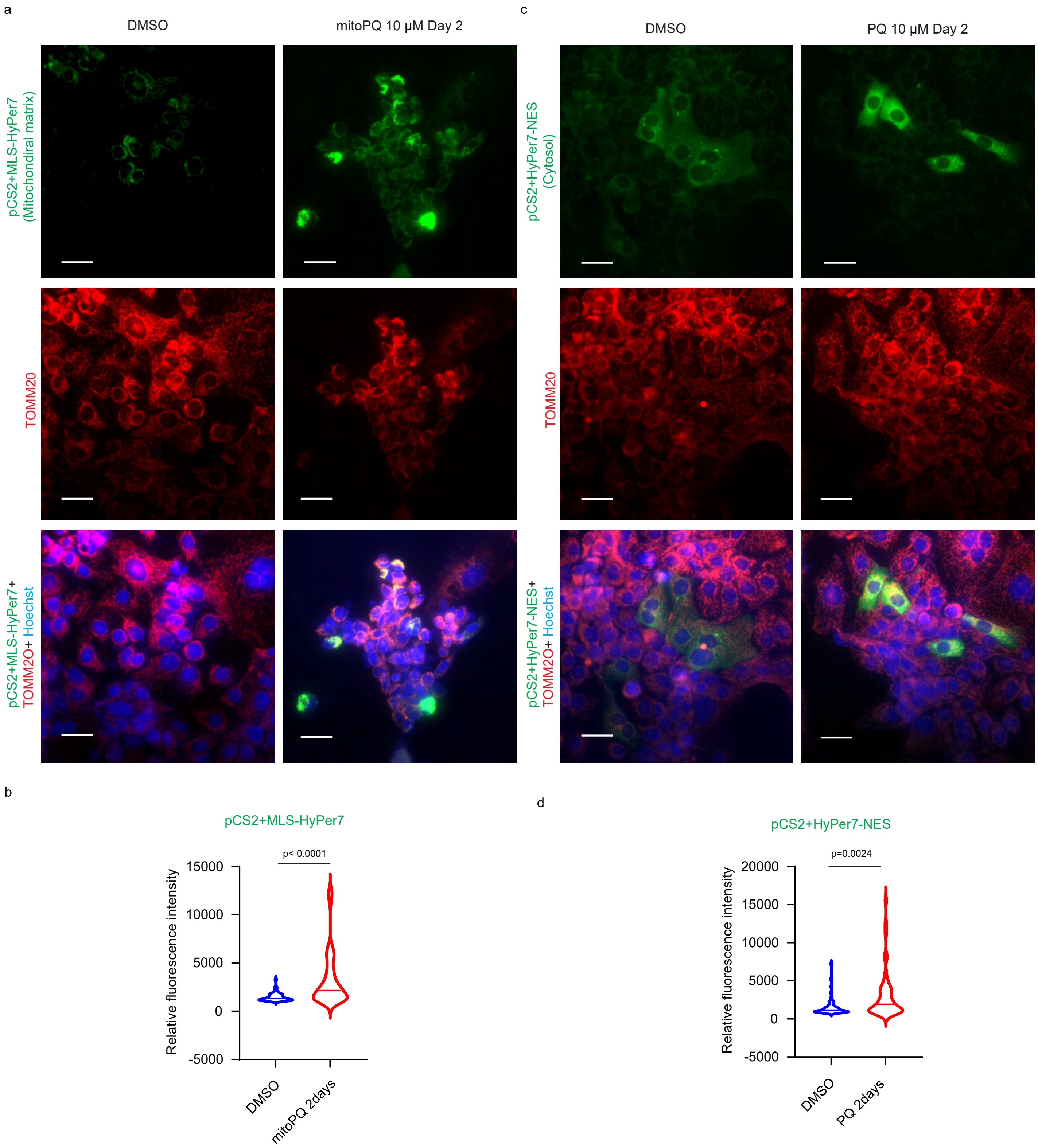
a
DMSO
mitoPQ 10 μM Day 2
pCS2+MLS-HyPer7
(Mitochondiral matrix)
TOMM20
pCS2+MLS-HyPer7+
TOMM2O+ Hoechst
c
DMSO
PQ 10 μM Day 2
pCS2+HyPer7-NES
(Cytosol)
TOMM20
pCS2+HyPer7-NES+
TOMM2O+ Hoechst
b
pCS2+MLS-HyPer7
p< 0.0001
Relative fluorescence intensity
15000
10000
5000
0
-5000
DMSO
mitoPQ 2days
d
pCS2+HyPer7-NES
p=0.0024
Relative fluorescence intensity
20000
15000
10000
5000
0
-5000
DMSO
PQ 2days

**Supplementary Figure 8. Two pH-stable GFP probes for hydrogen peroxide monitor mitochondrial localized-ROS and cytosol localized-ROS.** a) PATC66 cells were transfected with the hydrogen peroxide indicator pCS2+MLS-Hyper7, which is localized to the mitochondrial matrix, and treated with 10 μM Mito-PQ or Paraquat (PQ) for 2 days. b) Quantification of fluorescence intensity in (a). At least 50 cells with positive green fluorescence were calculated for each group. c) PATC66 cells were transfected with the hydrogen peroxide indicator pCS2+HyPer7-NES, which is localized to the cytoplasm, and treated with 10 μM Mito-PQ or PQ for 2 days. d) Quantification of fluorescence intensity in (c). At least 50 cells with positive green fluorescence were calculated for each group. For (a) and (c), mitochondria were identified with immunohistochemistry against the mitochondrial outer membrane protein, TOMM20. Nuclei were stained by Hoechst 33342. Scale bar, 30μm. Statistical analysis by two-tailed Students' unpaired t test with significance indicated (b, d). Source data are provided as a Source Data file.

a

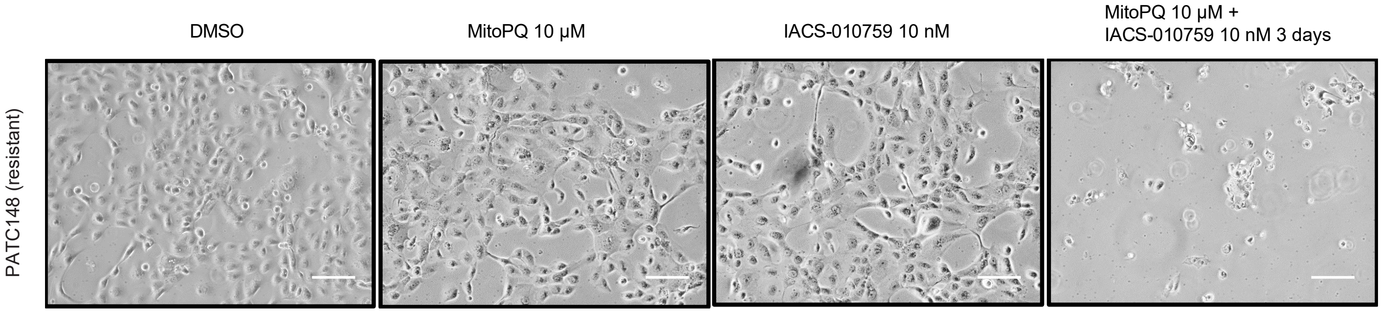
DMSO
MitoPQ 10 µM
IACS-010759 10 nM
MitoPQ 10 µM +
IACS-010759 10 nM 3 days
PATC148 (resistant)

b

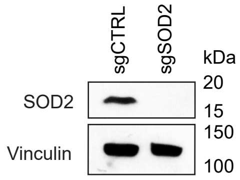
sgCTRL
sgSOD2
kDa
20
15
150
100
SOD2
Vinculin

**Supplementary Figure 9. Mitochondrial ROS induction increases cell sensitivity to IACS-010759.** a) Representative bright field images taken after a 3 day-treatment with 10 nM IACS-010759 with or without 10 µM MitoPQ in PATC148. Scale bar, 50 µm. 3 independent replicates had been done with similar results. b) Western blot with SOD2 and vinculin antibodies in PATC124 cells infected with control sgRNA (sgCTRL) or sgRNA targeting SOD2 (sgSOD2). 2 independent replicates had been done with similar results. Source data are provided as a Source Data file.

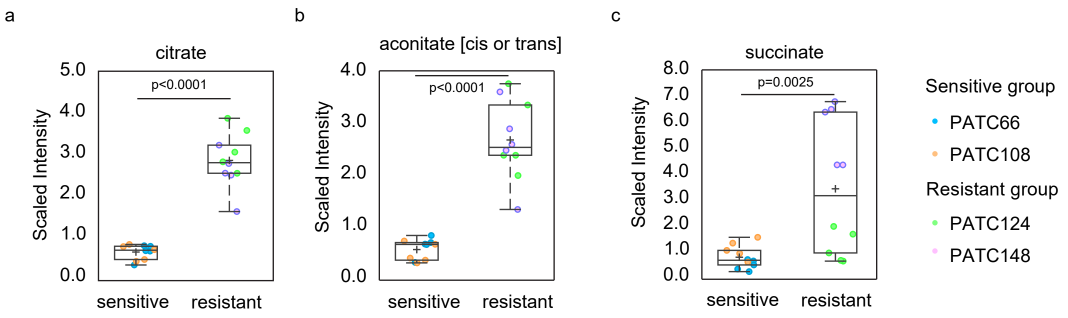
a
citrate
p<0.0001
Scaled Intensity
5.0
4.0
3.0
2.0
1.0
0.0
sensitive
resistant
b
aconitate [cis or trans]
p<0.0001
Scaled Intensity
4.0
3.0
2.0
1.0
0.0
sensitive
resistant
c
succinate
p=0.0025
Scaled Intensity
8.0
7.0
6.0
5.0
4.0
3.0
2.0
1.0
0.0
sensitive
resistant
Sensitive group
PATC66
PATC108
Resistant group
PATC124
PATC148

**Supplementary Figure 10. Higher TCA cycle components in resistant group. a-c**) Relative amounts of citrate (a), aconitate [cis or trans] (b), succinate (c) in sensitive (PATC66/108) and resistant (PATC124/148) lines. The boxes range from 25$^{th}$ and the 75$^{th}$ percentiles; Horizontal lines within box denote median value, and crosses denote mean value; whiskers denote error bars with 5% and 95% percentiles. 5 biologically independent replicates per cell line. Data represent mean ± S.D between sensitive group (2 cell lines) and resistant group (2 cell lines). Statistical analysis by two-tailed Students' unpaired t test with significance indicated (a-c). Source data are provided as a Source Data file.

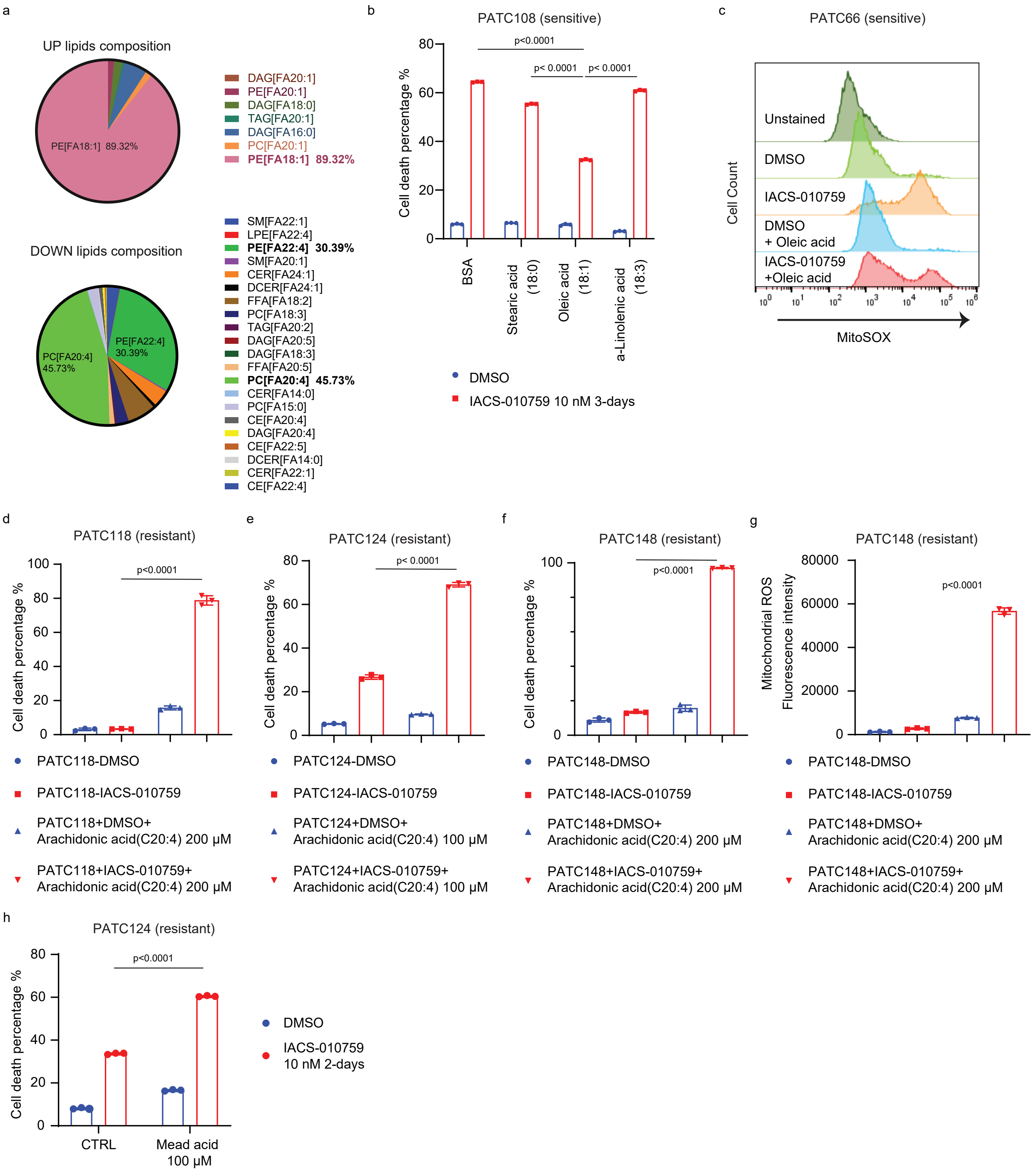

a
UP lipids composition
PE[FA18:1] 89.32%
DAG[FA20:1]
PE[FA20:1]
DAG[FA18:0]
TAG[FA20:1]
DAG[FA16:0]
PC[FA20:1]
PE[FA18:1] 89.32%
DOWN lipids composition
PC[FA20:4] 45.73%
PE[FA22:4] 30.39%
SM[FA22:1]
LPE[FA22:4]
PE[FA22:4] 30.39%
SM[FA20:1]
CER[FA24:1]
DCER[FA24:1]
FFA[FA18:2]
PC[FA18:3]
TAG[FA20:2]
DAG[FA20:5]
DAG[FA18:3]
FFA[FA20:5]
PC[FA20:4] 45.73%
CER[FA14:0]
PC[FA15:0]
CE[FA20:4]
DAG[FA20:4]
CE[FA22:5]
DCER[FA14:0]
CER[FA22:1]
CE[FA22:4]
b
PATC108 (sensitive)
Cell death percentage %
p<0.0001
p< 0.0001
p< 0.0001
BSA
Stearic acid (18:0)
Oleic acid (18:1)
a-Linolenic acid (18:3)
DMSO
IACS-010759 10 nM 3-days
c
PATC66 (sensitive)
Cell Count
Unstained
DMSO
IACS-010759
DMSO + Oleic acid
IACS-010759 +Oleic acid
MitoSOX
d
PATC118 (resistant)
Cell death percentage %
p<0.0001
PATC118-DMSO
PATC118-IACS-010759
PATC118+DMSO+ Arachidonic acid(C20:4) 200 μM
PATC118+IACS-010759+ Arachidonic acid(C20:4) 200 μM
e
PATC124 (resistant)
Cell death percentage %
p< 0.0001
PATC124-DMSO
PATC124-IACS-010759
PATC124+DMSO+ Arachidonic acid(C20:4) 100 μM
PATC124+IACS-010759+ Arachidonic acid(C20:4) 100 μM
f
PATC148 (resistant)
Cell death percentage %
p<0.0001
PATC148-DMSO
PATC148-IACS-010759
PATC148+DMSO+ Arachidonic acid(C20:4) 200 μM
PATC148+IACS-010759+ Arachidonic acid(C20:4) 200 μM
g
PATC148 (resistant)
Mitochondrial ROS Fluorescence intensity
p<0.0001
PATC148-DMSO
PATC148-IACS-010759
PATC148+DMSO+ Arachidonic acid(C20:4) 200 μM
PATC148+IACS-010759+ Arachidonic acid(C20:4) 200 μM
h
PATC124 (resistant)
Cell death percentage %
p<0.0001
CTRL
Mead acid 100 μM
DMSO
IACS-010759 10 nM 2-days

**Supplementary Figure 11. MUFAs and PUFAs exhibit opposite contribution to cell survival and ROS homeostasis upon mitochondrial complex I inhibition.** a) Composition of lipid species among lipids that are differentially presented in resistant versus sensitive cells. b) Cell death detected by propidium iodide staining in PATC108 cells upon DMSO or 10 nM IACS-010759 treatment in the presence of either 200 μM stearic acid (C18:0), oleic acid (C18:1) or -Linolenic acid (C18:3), respectively. Data represent mean ± S.D of 3 biologically independent replicates. c) Mitochondrial ROS detected by MitoSOX staining in PATC66 cells after 3 days of treatment with DMSO or 10 nM IACS-010759 in the presence or absence of 200 μM oleic acid. d-f) Cell death in PATC118 (d), PATC124 (e), and PATC148 (f) after 3-days of treatment with DMSO or 10 nM IACS-010759, in the presence or absence of PUFA (arachidonic acid C20:4) at the indicated concentration. Data represent mean ± S.D of 3 biologically independent replicates. g) Mitochondrial ROS were detected in PATC148 cells upon 3-days treatment with DMSO or 10 nM IACS-010759, in the presence or absence of 200 μM PUFA (arachidonic acid C20:4). Data represent mean ± S.D of 3 biologically independent replicates. h) PATC124 cells were treated with DMSO or 10nM IACS-010759, in the presence or absence of 100 μM mead acid, for 2-days. Propidium iodide (PI) stain was detected for cell death events by flowcytometry. Data represent mean ± S.D of 3 biologically independent replicates. Statistical analysis by two-tailed Students' unpaired t test with significance indicated (b, d-h). Source data are provided as a Source Data file.

a

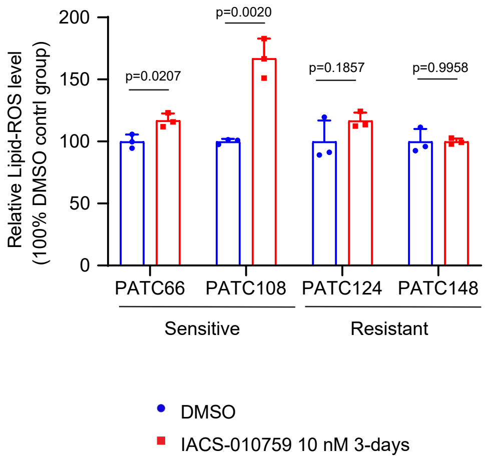
Relative Lipid-ROS level
(100% DMSO contrl group)
200
150
100
50
0
p=0.0207
p=0.0020
p=0.1857
p=0.9958
PATC66
PATC108
PATC124
PATC148
Sensitive
Resistant
DMSO
IACS-010759 10 nM 3-days

b

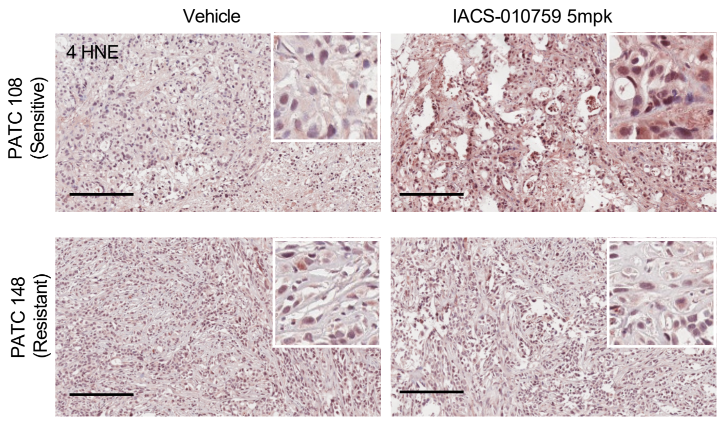
Vehicle
IACS-010759 5mpk
4 HNE
PATC 108
(Sensitive)
PATC 148
(Resistant)

c

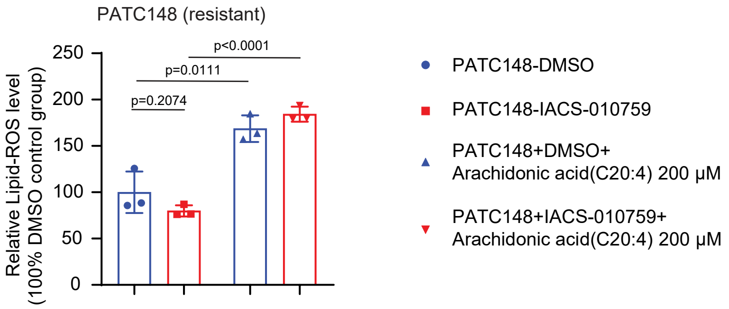
PATC148 (resistant)
Relative Lipid-ROS level
(100% DMSO control group)
250
200
150
100
50
0
p<0.0001
p=0.0111
p=0.2074
PATC148-DMSO
PATC148-IACS-010759
PATC148+DMSO+
Arachidonic acid(C20:4) 200 μM
PATC148+IACS-010759+
Arachidonic acid(C20:4) 200 μM

**Supplementary Figure 12. Complex I inhibition induces lipid peroxidation in cells sensitive to IACS-010759 treatment. a)** Lipid peroxidation were detected by BODIPY 581/591 C11 in complex I inhibitor-sensitive (PATC66/108) and resistant (PATC124/148) cells treated with DMSO or 10 nM IACS-010759 for 3-days. **b)** Representative 4 HNE stain in biopsies from sensitive xenograft tumor (PATC108) and resistant tumors (PATC148) in present and absent of IACS-010759 with 5 mpk, showed lipid peroxidation in vivo. Scale bar, 200 μm. c) Lipid peroxidation were detected by BODIPY 581/591 C11 in complex I inhibitor resistant PATC148 cells treated with DMSO or 10 nM IACS-010759, with or without 200 μM PUFA C20:4, for 2-days. Data represent mean ± S.D of 3 biologically independent replicates (a, c). Statistical analysis by two-tailed Students' unpaired t test with significance indicated (a, c). Source data are provided as a Source Data file.

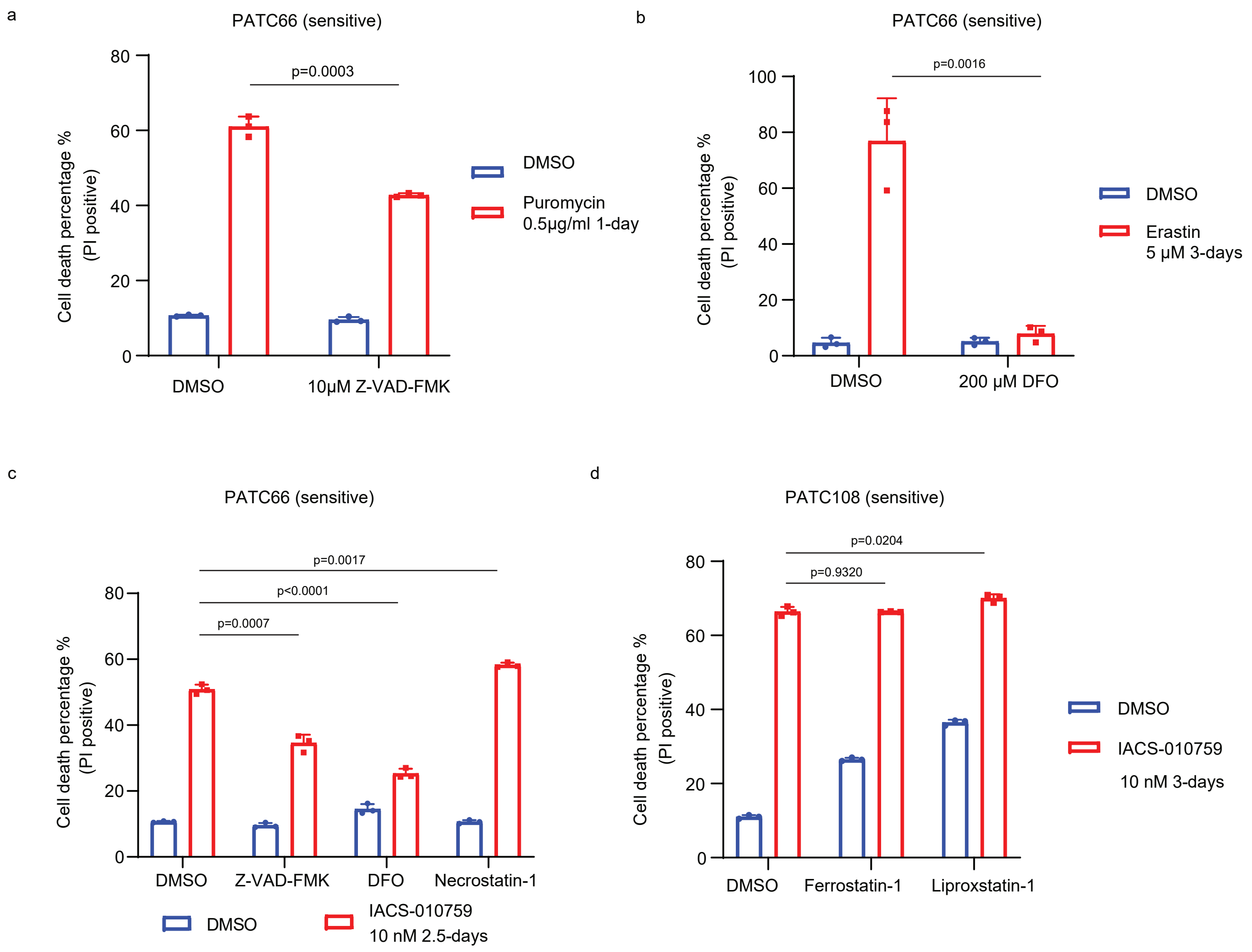
a
PATC66 (sensitive)
p=0.0003
Cell death percentage %
(PI positive)
80
60
40
20
0
DMSO
10μM Z-VAD-FMK
DMSO
Puromycin
0.5μg/ml 1-day
b
PATC66 (sensitive)
p=0.0016
Cell death percentage %
(PI positive)
100
80
60
40
20
0
DMSO
200 μM DFO
DMSO
Erastin
5 μM 3-days
c
PATC66 (sensitive)
p=0.0017
p<0.0001
p=0.0007
Cell death percentage %
(PI positive)
80
60
40
20
0
DMSO
Z-VAD-FMK
DFO
Necrostatin-1
DMSO
IACS-010759
10 nM 2.5-days
d
PATC108 (sensitive)
p=0.0204
p=0.9320
Cell death percentage %
(PI positive)
80
60
40
20
0
DMSO
Ferrostatin-1
Liproxstatin-1
DMSO
IACS-010759
10 nM 3-days

**Supplementary Figure 13. A deep dissection of cell death mechanism induced by IACS-010759.** a-c) PATC66 cells were treated with 0.5 μg/ml puromycin, with or without 10 μM Z-VAD-FMK (a); 5 μM Erastin, with or without 200 μM DFO for 3-days (b), DMSO or 10 nM IACS-010759, with or without 10 μM Z-VAD-FMK, 200 μM DFO or 40 μM Necrostatin-1 for 2.5-days (c). d) PATC108 cells were treated with DMSO or 10 nM IACS-010759, with or without 10 μM Ferrostatin-1/ Liproxstatin-1 for 3-days. Flow cytometry showed PI positive cell death events. Data represent mean ± S.D of 3 biologically independent replicates (a-d). Statistical analysis by two-tailed Students' unpaired t test with significance indicated (a-d). Source data are provided as a Source Data file.

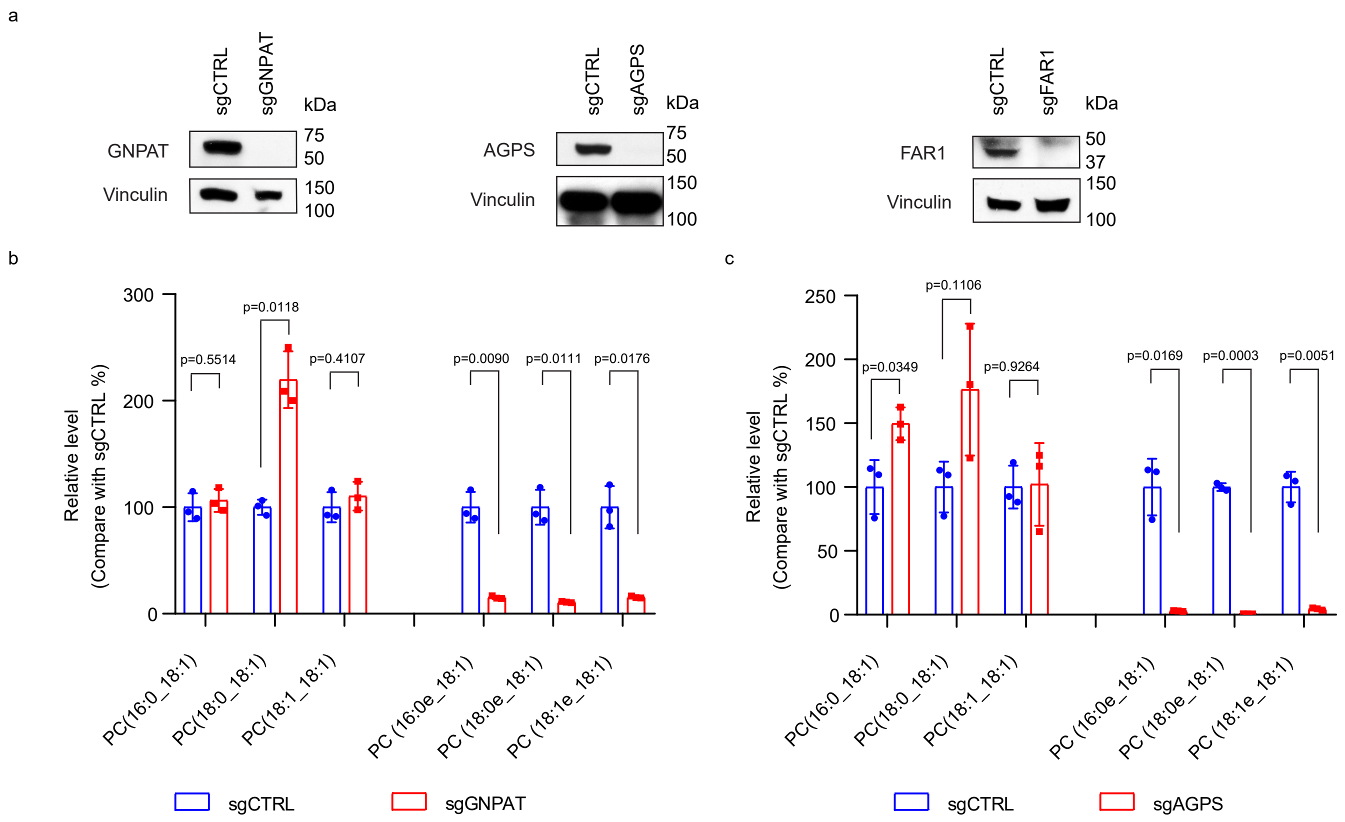

a
sgCTRL
sgGNPAT
kDa
GNPAT
75
50
Vinculin
150
100
sgCTRL
sgAGPS
kDa
AGPS
75
50
Vinculin
150
100
sgCTRL
sgFAR1
kDa
FAR1
50
37
Vinculin
150
100
b
Relative level
(Compare with sgCTRL %)
300
200
100
0
p=0.5514
p=0.0118
p=0.4107
p=0.0090
p=0.0111
p=0.0176
PC(16:0_18:1)
PC(18:0_18:1)
PC(18:1_18:1)
PC (16:0e_18:1)
PC (18:0e_18:1)
PC (18:1e_18:1)
sgCTRL
sgGNPAT
c
Relative level
(Compare with sgCTRL %)
250
200
150
100
50
0
p=0.0349
p=0.1106
p=0.9264
p=0.0169
p=0.0003
p=0.0051
PC(16:0_18:1)
PC(18:0_18:1)
PC(18:1_18:1)
PC (16:0e_18:1)
PC (18:0e_18:1)
PC (18:1e_18:1)
sgCTRL
sgAGPS

**Supplementary Figure 14. Blocking de novo ether phospholipid synthesis by knocking out key ether lipids generation enzymes.** a) Western blot of GNPAT, AGPS, and FAR1 in PATC124 cells infected with corresponding sgRNAs. b-c) Relative levels of indicated phospholipid or ether phospholipid in PATC124 cells infected with control sgRNA (sgCTRL), sgRNA targeting GNPAT (sgGNPAT) (b), or AGPS (sgAGPS) (c). Data represent mean ± S.D of 3 biologically independent replicates (b, c). Statistical analysis by two-tailed Students' unpaired t test with significance indicated (b, c). Source data are provided as a Source Data file.

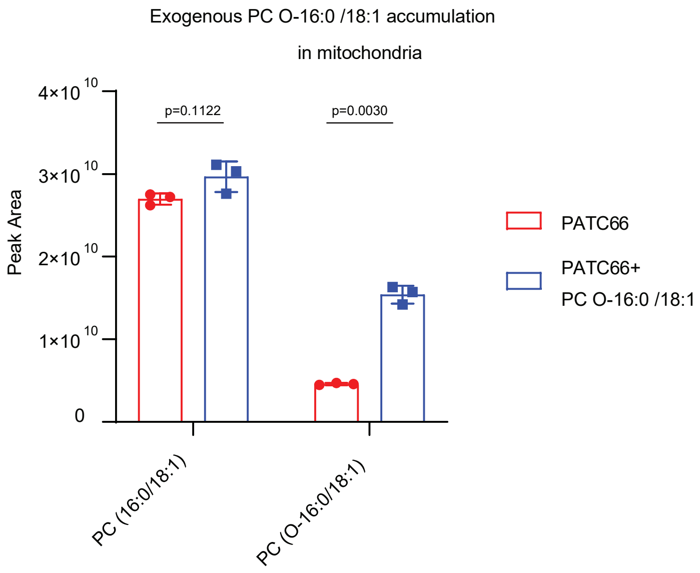

Exogenous PC O-16:0 /18:1 accumulation
in mitochondria
Peak Area
4×10 10
3×10 10
2×10 10
1×10 10
0
p=0.1122
p=0.0030
PC (16:0/18:1)
PC (O-16:0/18:1)
PATC66
PATC66+
PC O-16:0 /18:1

**Supplementary Figure 15. Supplementation with C18:1-linked ether lipids (O-C16-18:1 PC) induce accumulation in mitochondria.** a) After incubated with or without 100 μM exogenous (O-C16-18:1 PC) for 12 hours, mitochondria from PATC66 were extracted and detected for enrichment of total PC (16:0-18:1) and ether-PC (O-16:0-18:1). Data normalized by extracted mitochondrial mass. Data represent mean ± S.D of 3 biologically independent replicates. Statistical analysis by two-tailed Students' unpaired t test with significance indicated. Source data are provided as a Source Data file.

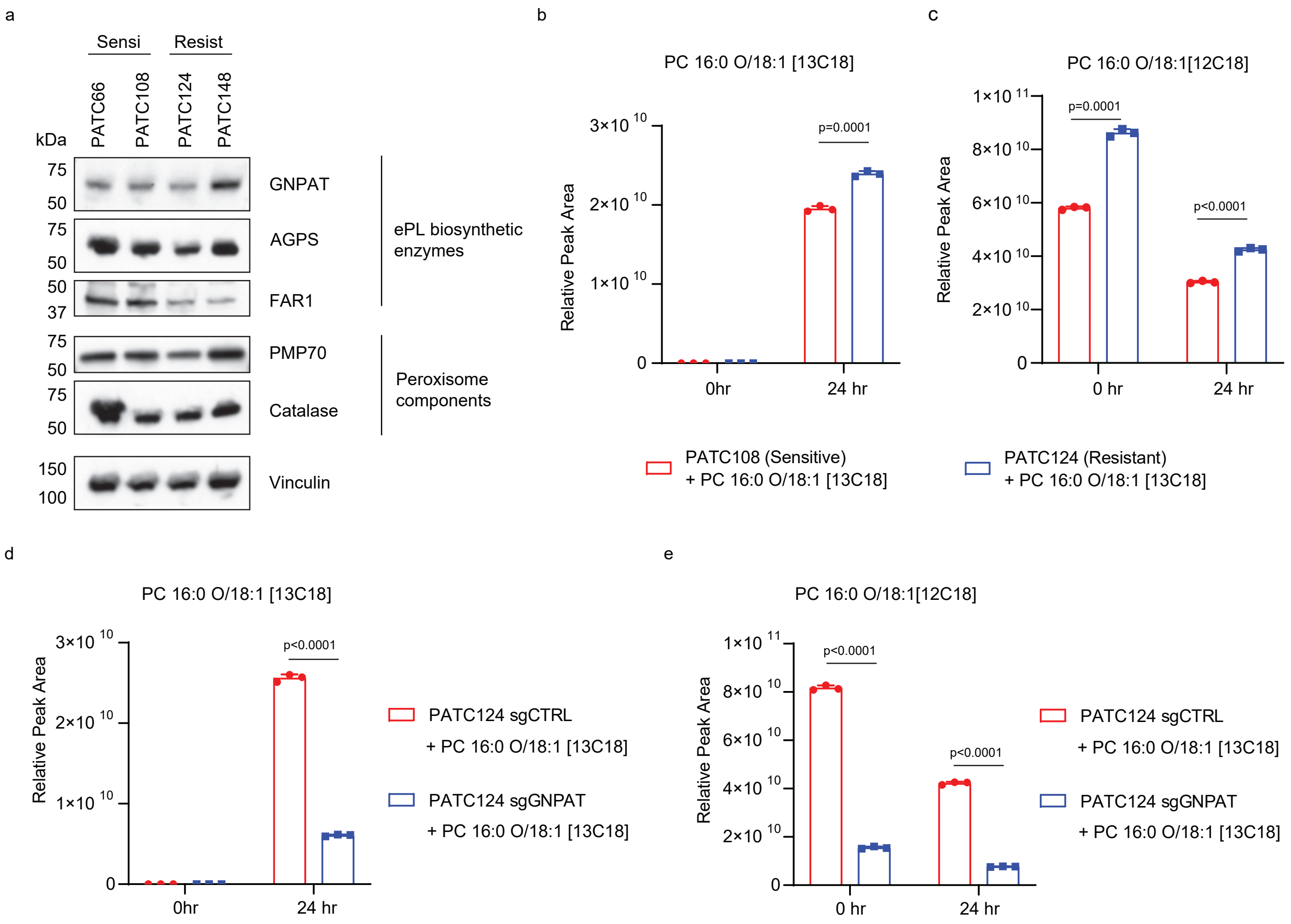
a
Sensi
Resist
PATC66
PATC108
PATC124
PATC148
kDa
75
50
GNPAT
75
50
AGPS
50
37
FAR1
75
50
PMP70
75
50
Catalase
150
100
Vinculin
ePL biosynthetic enzymes
Peroxisome components
b
PC 16:0 O/18:1 [13C18]
Relative Peak Area
p=0.0001
0hr
24 hr
c
PC 16:0 O/18:1[12C18]
Relative Peak Area
p=0.0001
p<0.0001
0 hr
24 hr
PATC108 (Sensitive)
+ PC 16:0 O/18:1 [13C18]
PATC124 (Resistant)
+ PC 16:0 O/18:1 [13C18]
d
PC 16:0 O/18:1 [13C18]
Relative Peak Area
p<0.0001
0hr
24 hr
PATC124 sgCTRL
+ PC 16:0 O/18:1 [13C18]
PATC124 sgGNPAT
+ PC 16:0 O/18:1 [13C18]
e
PC 16:0 O/18:1[12C18]
Relative Peak Area
p<0.0001
p<0.0001
0 hr
24 hr
PATC124 sgCTRL
+ PC 16:0 O/18:1 [13C18]
PATC124 sgGNPAT
+ PC 16:0 O/18:1 [13C18]

**Supplementary Figure 16. Peroxisome activities comparation between sensitive and resistant lines.** a) ether lipids biosynthetic enzymes (GNPAT, AGPS, FAR1) and peroxisome components (PMP70, catalase) comparation by western blotting. **b-e**) PATC108 / 124 cells **(b-c)** and PATC124 cells infected with control sgRNA (sgCTRL) or sgRNA targeting *GNPAT* (sgGNPAT) **(d-e)** were incubated with labeled oleic acid with 200 μM $^{13}C$ (Oleic acid-$^{13}C_{18}$) for 1-day. The labeled / unlabeled and ether lipids (PC 16:0 O/18:1[$^{13}C18$], or PC 16:0 O/18:1[$^{12}C18$],) were detected and normalized by harvested cell number. Data represent mean ± S.D of 3 biologically independent replicates (b-e). Statistical analysis by two-tailed Students' unpaired t test with significance indicated (b-e). Source data are provided as a Source Data file.

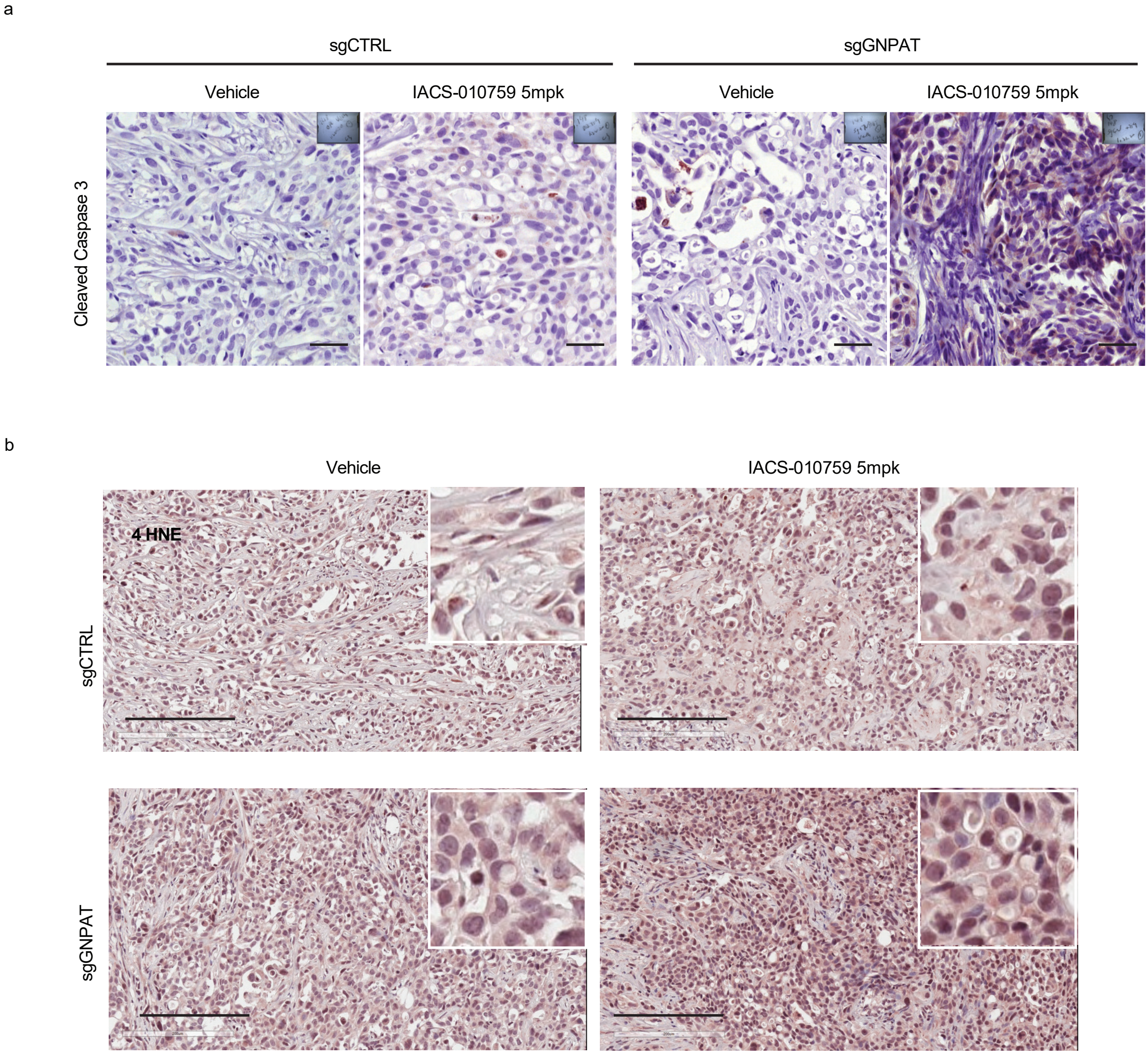

a
sgCTRL
sgGNPAT
Vehicle
IACS-010759 5mpk
Vehicle
IACS-010759 5mpk
Cleaved Caspase 3
b
Vehicle
IACS-010759 5mpk
4 HNE
sgCTRL
sgGNPAT

**Supplementary Figure 17. GNPAT-deleted tumors with IACS-010759 increased cell death and lipids peroxidation.** a) Representative IHC staining for apoptotic marker cleaved-Caspase 3 in sgCTRL/sgGNPAT PATC124 subcutaneous tumors treated with vehicle or 5 mg/kg IACS-010759. Scale bar, 50 μm. The experiment had been repeated individually 3 times with similar results. b) Representative 4 HNE stain in biopsies from sgCTRL/sgGNPAT PATC124 xenograft tumors, in present and absent of IACS-010759 with 5 mg/kg, showed lipid peroxidation in vivo. Scale bar, 200 μm. 3 independent replicates had been done with similar results.

a

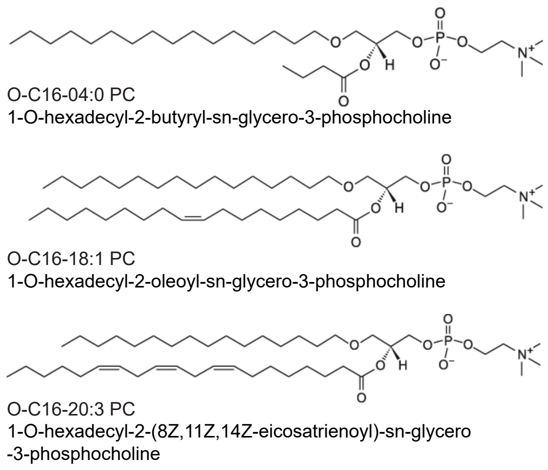
O-C16-04:0 PC
1-O-hexadecyl-2-butyryl-sn-glycero-3-phosphocholine
O-C16-18:1 PC
1-O-hexadecyl-2-oleoyl-sn-glycero-3-phosphocholine
O-C16-20:3 PC
1-O-hexadecyl-2-(8Z,11Z,14Z-eicosatrienoyl)-sn-glycero
-3-phosphocholine

b

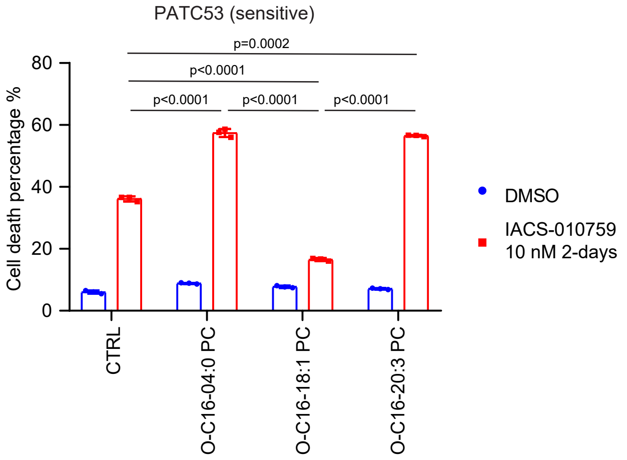
PATC53 (sensitive)
p=0.0002
p<0.0001
p<0.0001
p<0.0001
p<0.0001
Cell death percentage %
CTRL
O-C16-04:0 PC
O-C16-18:1 PC
O-C16-20:3 PC
DMSO
IACS-010759
10 nM 2-days

c

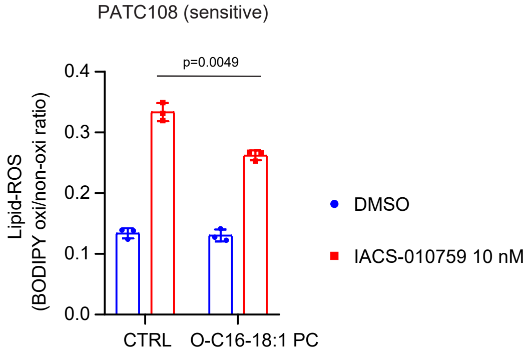
PATC108 (sensitive)
p=0.0049
Lipid-ROS
(BODIPY oxi/non-oxi ratio)
CTRL
O-C16-18:1 PC
DMSO
IACS-010759 10 nM

**Supplementary Figure 18. MUFA-linked ether phosphatidylcholine promotes resistance to mitochondrial complex I inhibition. a)** Structural formula of SFA (O-C16-04:0 PC), MUFA (O-C16-18:1 PC), and PUFA (O-C16-20:3 PC)-linked ether phosphatidylcholine. **b)** Cell viability was detected by propidium iodide staining and flow cytometry in PATC53 cells upon treatment with DMSO or 10 nM IACS-010759 in the presence of indicated ether phosphatidylcholine. Data represent mean ± S.D of 3 biologically independent replicates. **c)** Lipid ROS were detected by BODIPY 581/591 C11 after a 3-day treatment with DMSO or 10 nM IACS-010759 in PATC108 cells grown in the presence or absence of 100 µM O-C16-18:1 PC. Data represent mean ± S.D of 3 biologically independent replicates. Statistical analysis by two-tailed Students' unpaired t test with significance indicated (b, c). Source data are provided as a Source Data file.

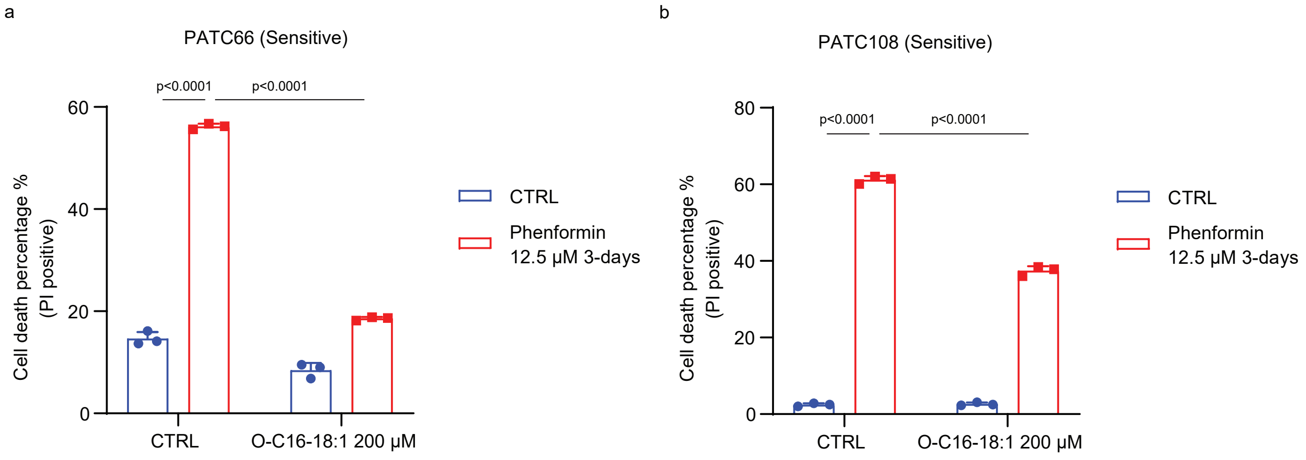
a
PATC66 (Sensitive)
p<0.0001
p<0.0001
Cell death percentage %
(PI positive)
60
40
20
0
CTRL
O-C16-18:1 200 μM
CTRL
Phenformin
12.5 μM 3-days
b
PATC108 (Sensitive)
p<0.0001
p<0.0001
Cell death percentage %
(PI positive)
80
60
40
20
0
CTRL
O-C16-18:1 200 μM
CTRL
Phenformin
12.5 μM 3-days

**Supplementary Figure 19. MUFA-linked ether phosphatidylcholine rescue cell death induced by mitochondrial complex I inhibitor phenformin**. PATC66 (a) and PATC108 (b) cells were treated with DMSO or 12.5 μM phenformin, in the presence or absence of 200 μM ether-MUFA O-C16-18:1, for 3-days. Propidium iodide (PI) stain was detected for cell death events by flowcytometry. Data represent mean ± S.D of 3 biologically independent replicates. Statistical analysis by two-tailed Students' unpaired t test with significance indicated (a, b). Source data are provided as a Source Data file.

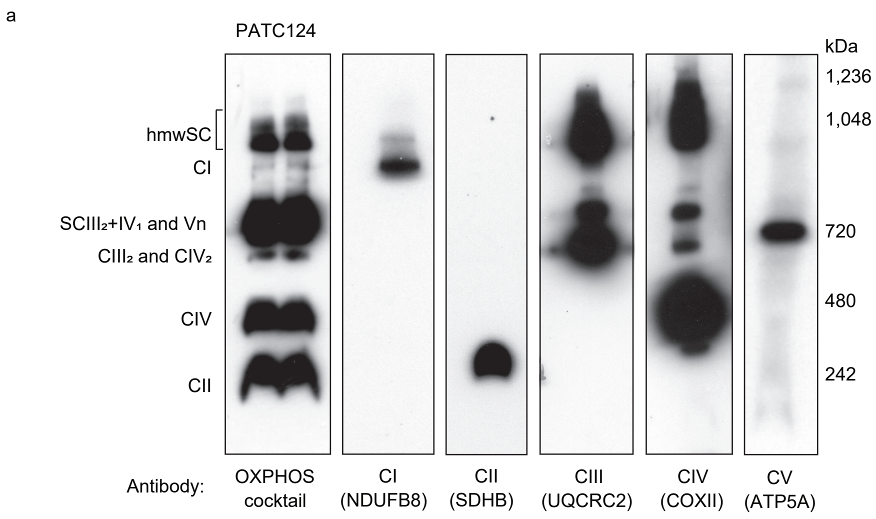
a
PATC124
kDa
1,236
1,048
720
480
242
hmwSC
CI
SCIII₂+IV₁ and Vn
CIII₂ and CIV₂
CIV
CII
Antibody:
OXPHOS cocktail
CI (NDUFB8)
CII (SDHB)
CIII (UQCRC2)
CIV (COXII)
CV (ATP5A)

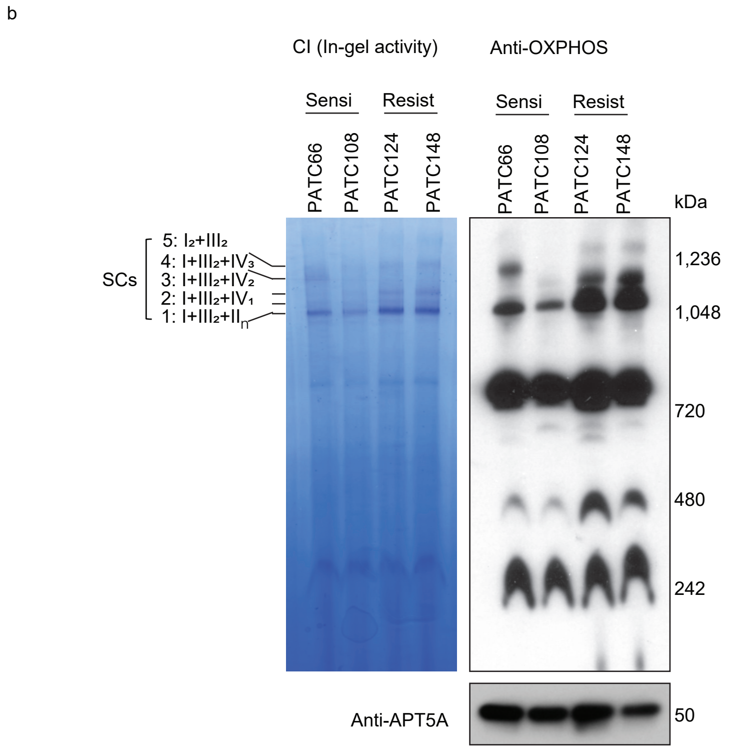
b
CI (In-gel activity)
Anti-OXPHOS
Sensi
Resist
PATC66
PATC108
PATC124
PATC148
kDa
SCs
5: I₂+III₂
4: I+III₂+IV₃
3: I+III₂+IV₂
2: I+III₂+IV₁
1: I+III₂+IIn
1,236
1,048
720
480
242
Anti-APT5A
50

**Supplementary Figure 20. Blue native PAGE and In-gel activity protocols identified mitochondrial supercomplexes.** a) Mitochondrial supercomplexes (SC) were extracted in PATC124 cells and six equal samples were run with blue native PAGE in the same gel. Sample was then transferred to one PVDF membrane and divided into six parts. the six membrane parts were incubated with either an anti-OXPHOS antibody cocktail, anti-NDUFB8 antibody (Complex I targeting), anti-SDHB antibody (Complex II targeting), anti-UQCRC2 antibody (Complex III targeting), anti-COXII antibody (Complex IV targeting), or anti-ATP5A antibody (Complex V targeting). The experiment had been repeated individually 2 times with similar results. b) mitochondrial supercomplexes (SC) activity and mass in sensitive (PATC66/108) and resistant (PATC124/148) cells. 100 μg mitochondria were extracted from sensitive lines (PATC66/108) and resistant lines (PATC124/148) for BN-PAGE gel running followed by incubation with complex I substrate NADH (0.1mg/ml) for 30min in room temperature. 10% acetic acid was used to stop reaction. Supercomplexes containing complex I were showed in BN-PAGE gel with purple bands. 50 μg mitochondria were extracted for BN-PAGE blot to detect SCs mass by anti-OXPHOS and anti-ATP5A antibodies incubation. 3 independent replicates had been done with similar results.

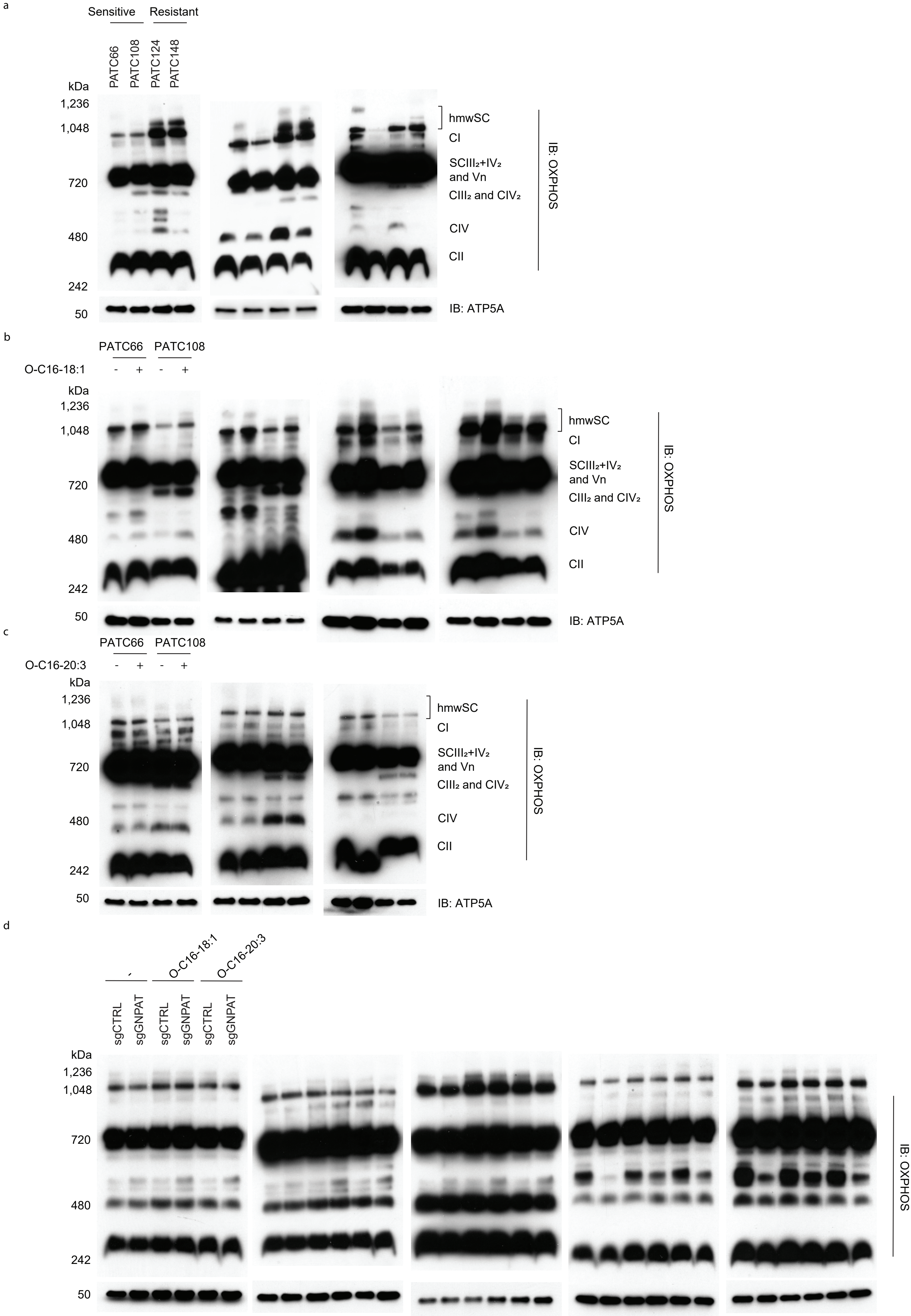

a
Sensitive
Resistant
PATC66
PATC108
PATC124
PATC148
kDa
1,236
1,048
720
480
242
50
hmwSC
CI
SCIII₂+IV₂
and Vn
CIII₂ and CIV₂
CIV
CII
IB: OXPHOS
IB: ATP5A
b
PATC66
PATC108
O-C16-18:1
- + - +
IB: OXPHOS
IB: ATP5A
c
PATC66
PATC108
O-C16-20:3
- + - +
IB: OXPHOS
IB: ATP5A
d
-
O-C16-18:1
O-C16-20:3
sgCTRL
sgGNPAT
IB: OXPHOS

**Supplementary Figure 21. MUFA-linked ether phosphatidylcholine promotes the formation of high-molecular-weight supercomplexes**. a) Mitochondrial supercomplexes (SC) in PATC66 / 108 / 124 / 148 cells as shown with BN-PAGE in three independent experiments. b) Mitochondrial supercomplexes in PATC66/108 cells grown in the presence or absence of 100 μM ether-MUFA (O-C16-18:1 phosphatidylcholin (PC)) for 24 hours. Four independent BN-PAGE results were shown. c) Miitochondrial supercomplexes in PATC66/108 cells grown in the presence or absence of 100 μM ether-PUFA (O-C16-20:3 phosphatidylcholin (PC)) for 24 hours. Three independent BN-PAGE results were shown. d) Miitochondrial supercomplexes in PATC124 cells infected with control sgRNA (sgCTRL) or sgRNA targeting *GNPAT* (sgGNPAT) in the presence or absence of 100 μM O-C16-18:1 PC or 100 μM ether-PUFA (O-C16-20:3 PC) for 24 hours. 5 independent BN-PAGE results were shown.

a

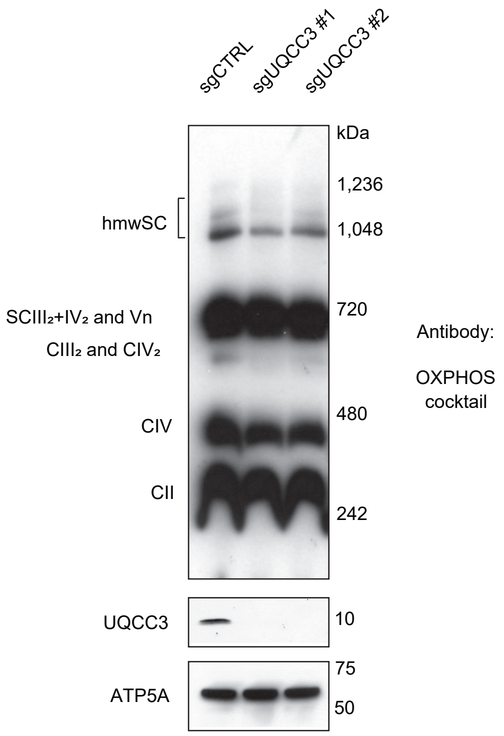

sgCTRL
sgUQCC3 #1
sgUQCC3 #2
kDa
1,236
1,048
720
480
242
hmwSC
SCIII₂+IV₂ and Vn
CIII₂ and CIV₂
CIV
CII
Antibody:
OXPHOS cocktail
UQCC3
10
ATP5A
75
50


b

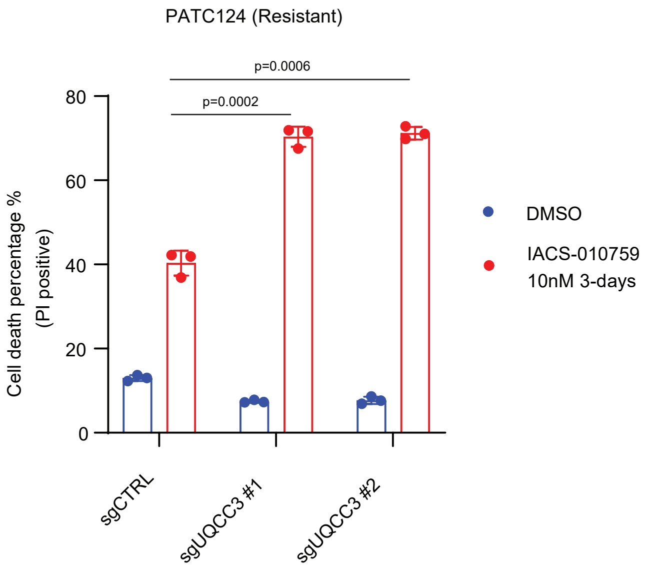

PATC124 (Resistant)
p=0.0006
p=0.0002
80
60
40
20
0
Cell death percentage %
(PI positive)
DMSO
IACS-010759
10nM 3-days
sgCTRL
sgUQCC3 #1
sgUQCC3 #2

**Supplementary Figure 22. Decrease to SCs assembly by knocking out UQCC3 sensitize to complex I inhibition.** a) SCs samples were extracted and components were detected in control (sgCTRL) and UQCC3 deleted PATC124 cells. b) control (sgCTRL) and UQCC3 deleted PATC124 cells were treated with DMSO or 10nM IACS-010759 for 3-days. Cell viability were detected by PI stain. Data represent mean ± S.D of 3 biologically independent replicates. Statistical analysis by two-tailed Students' unpaired t test with significance indicated (b). Source data are provided as a Source Data file.

**Supplementary Table 1. IC50 for IACS-010759 in PDXs.**

| | **Sensitive** | | | **Resistant** | | |
|---|---|---|---|---|---|---|
| PATCs | PATC53 | PATC66 | PATC108 | PATC118 | PATC124 | PATC148 |
| IC50 (nmol/L)) | 6.7 | 5.1 | 7.1 | 13.8 | 13.5 | 20.2 |

**Supplementary Table 2. Main genetic mutation features in PDXs.**

| MODEL | SNPEFF_GENE_NAME | SNPEFF_AA_CHANGE | Sensitivity to complex I |
|---|---|---|---|
| PDX-PDAC-053 | KRAS | p. G12D | Sensitive PDXs |
| PDX-PDAC-053 | TP53 | p. R306* | |
| PDX-PDAC-066 | KRAS | p. G12D | |
| PDX-PDAC-066 | TP53 | p. Y234* | |
| PDX-PDAC-066 | CDKN2A | p. Y44fs | |
| PDX-PDAC-108 | KRAS | p. G12D | |
| PDX-PDAC-108 | TP53 | p. R175H | |
| PDX-PDAC-118 | KRAS | p. Q61H | Resistant PDXs |
| PDX-PDAC-118 | TP53 | p. E224D | |
| PDX-PDAC-118 | CDKN2A | NA | |
| PDX-PDAC-124 | KRAS | p. G12D | |
| PDX-PDAC-124 | TP53 | p. R333fs | |
| PDX-PDAC-124 | SMAD4 | NA | |
| PDX-PDAC-148 | KRAS | p. G12D | |
| PDX-PDAC-148 | TP53 | p. F134L | |
