# Supplementary material for: Ether phospholipids are required for mitochondrial reactive oxygen species homeostasis
Source: Nat Commun. 2023 Apr 17;14:2194. doi: 10.1038/s41467-023-37924-9 (PMC10110566; doi:10.1038/s41467-023-37924-9)
Supplement: Supplementary file 3 — Reporting Summary [file 41467_2023_37924_MOESM3_ESM.pdf]

nature portfolio

Corresponding author(s): Giulio Draetta

Last updated by author(s): Dec 12, 2022

Reporting Summary

Nature Portfolio wishes to improve the reproducibility of the work that we publish. This form provides structure for consistency and transparency in reporting. For further information on Nature Portfolio policies, see our Editorial Policies and the Editorial Policy Checklist.

Statistics

For all statistical analyses, confirm that the following items are present in the figure legend, table legend, main text, or Methods section.

n/a	Confirmed	
☐	☒	The exact sample size (*n*) for each experimental group/condition, given as a discrete number and unit of measurement
☐	☒	A statement on whether measurements were taken from distinct samples or whether the same sample was measured repeatedly
☐	☒	The statistical test(s) used AND whether they are one- or two-sided<br>*Only common tests should be described solely by name; describe more complex techniques in the Methods section.*
☒	☐	A description of all covariates tested
☒	☐	A description of any assumptions or corrections, such as tests of normality and adjustment for multiple comparisons
☐	☒	A full description of the statistical parameters including central tendency (e.g. means) or other basic estimates (e.g. regression coefficient) AND variation (e.g. standard deviation) or associated estimates of uncertainty (e.g. confidence intervals)
☐	☒	For null hypothesis testing, the test statistic (e.g. *F*, *t*, *r*) with confidence intervals, effect sizes, degrees of freedom and *P* value noted<br>*Give P values as exact values whenever suitable.*
☒	☐	For Bayesian analysis, information on the choice of priors and Markov chain Monte Carlo settings
☒	☐	For hierarchical and complex designs, identification of the appropriate level for tests and full reporting of outcomes
☒	☐	Estimates of effect sizes (e.g. Cohen's *d*, Pearson's *r*), indicating how they were calculated

Our web collection on statistics for biologists contains articles on many of the points above.

Software and code

Policy information about availability of computer code

Data collection	The sgRNAs were designed using CRISPick website software (https://portals.broadinstitute.org/gppx/crispick/public) supported by BROAD Institute.
Data analysis	We used GraphPad Prism 9 software for statistics analysis and figures generation; FlowJo V10.6.2 was used for Flow Cytometry analysis; Image J (version 1.8.0) was used for western blotting data analysis and quantification of immunofluorescence signal. Lipidomics data analysis was performed with Thermo Scientific LipidSearch software (version 4.2.23) and TraceFinder software (version 5.1). Statistical analyses were conducted using R (version 3.6.0) .

For manuscripts utilizing custom algorithms or software that are central to the research but not yet described in published literature, software must be made available to editors and reviewers. We strongly encourage code deposition in a community repository (e.g. GitHub). See the Nature Portfolio guidelines for submitting code & software for further information.

Data

Policy information about availability of data

All manuscripts must include a data availability statement. This statement should provide the following information, where applicable:

- Accession codes, unique identifiers, or web links for publicly available datasets
- A description of any restrictions on data availability
- For clinical datasets or third party data, please ensure that the statement adheres to our policy

There are no restrictions on data availability for all main figures and supplementary figures.

Human research participants

Policy information about studies involving human research participants and Sex and Gender in Research.

Reporting on sex and gender	N/A
Population characteristics	N/A
Recruitment	N/A
Ethics oversight	N/A

Note that full information on the approval of the study protocol must also be provided in the manuscript.

Field-specific reporting

Please select the one below that is the best fit for your research. If you are not sure, read the appropriate sections before making your selection.

[x] Life sciences [ ] Behavioural & social sciences [ ] Ecological, evolutionary & environmental sciences

For a reference copy of the document with all sections, see nature.com/documents/nr-reporting-summary-flat.pdf

Life sciences study design

All studies must disclose on these points even when the disclosure is negative.

Sample size	1, For in vitro study, sample size was determined by reported literature (Nature.2014 Oct 30;514(7524):628-32), manufacturer's protocol, and previous outcomes, and stated for each experiment in the figure legends. In general, we used at lease 3 technical replicates to make sure minimal technical variations for all the experiments and made statistical differences with 3-5 independents experiments.<br>2, For in vivo study, samples size was determined by previous experiments (Nature.2014 Oct 30;514(7524):628-32). Based on our prior experience, we estimated that 5 mice per group would be enough to have statistically difference. Through the project, 5-10 mice (6-8 weeks with similar weight) was separately used for individual groups.
Data exclusions	No data was excluded.
Replication	Multiple independent replicates were included for related experiments, including cell culture, treatment, sample harvest, western blotting, flow cytometry, immunofluorescence staining etc. Each experiment was repeated at least twice. For each experiment, all the replicates showed similar results.
Randomization	6-8 weeks nude mice were randomly implanted with related PDX cells. When the subcutaneous tumors grow enough, only the mice whose tumor volume reached between 150-200 mm3 were selected and randomly divided into different groups as treatment Day 0. For cell experiments, cells were randomly located in incubators and randomly taken out for treatment. Other experimental units were equally distributed therefore randomization is inherent to our design.
Blinding	Western blotting was independently repeated by different investigators. When preparing cell samples, cell culturing, infection, and sample harvest were known by one researcher, However all data isolations and analysis such as knockout effect verification, cell availability detection were finished by another researcher to meet the blinding requirement. Immunofluorescence and IHC stain were prepared by our pathology core and were blinded to investigators. Tumor cell injection to mice and growing were responsible by one researcher, but tumor measurements and data analysis were finished by another independent researcher. Other experiments followed the same principle that the investigators related with data collection and analysis were always blinded to the samples preparation.

Reporting for specific materials, systems and methods

We require information from authors about some types of materials, experimental systems and methods used in many studies. Here, indicate whether each material, system or method listed is relevant to your study. If you are not sure if a list item applies to your research, read the appropriate section before selecting a response.

Materials & experimental systems

n/a	Involved in the study
☐	☒ Antibodies
☐	☒ Eukaryotic cell lines
☒	☐ Palaeontology and archaeology
☐	☒ Animals and other organisms
☒	☐ Clinical data
☒	☐ Dual use research of concern

Methods

n/a	Involved in the study
☒	☐ ChIP-seq
☐	☒ Flow cytometry
☒	☐ MRI-based neuroimaging

Antibodies

Antibodies used

anti-TOMM20 primary antibody (Proteintech, #11802-1-AP, 1:2000 dilution in PBS),anti-Cleaved Caspase 3 (Cell Signaling Technology, #9664L,1:1000 dilution), anti-Ki67 (Life Technologies, #MA514520,1:200 dilution), anti-SOD2/MnSOD (Abcam, #ab13533,1:2000 dilution), anti-GNPAT (Proteintech, #14931-1-AP, 1:1000 dilution), anti-AGPS (Sigma-Aldrich, #HPA030211, 1:1000 dilution), anti-FAR1 (Novus Biologicals, #NBP1-89847, 1:1000 dilution), total OXPHOS human WB antibody cocktail (Abcam, #ab110411, 1:1000 dilution), anti-ATP5A (Abcam, #ab14748, 1:2000 dilution), anti-vinculin (EMD Millipore, #05-386, clone V284, 1:3000 dilution), anti-mouse HRP-linked antibody (CellSignaling Technologies, 1:3000, #7076V), goat polyclonal secondary antibody (Goat anti-Rabbit Alexa Fluor 555, Fisher scientific, #PIA32732, 1:3000 dilution) , Goat anti-rabbit IgG ImmPRESS secondary antibody [HRP polymer] (Vector Biolabs MP-7451-50), anti-pimonidazole rabbit antisera (Hypoxyprobe, Inc. PAb2627AP, 1:50 dilution)

Validation

anti-TOMM20 primary antibody: Reactive with Human, Mouse, Rat, Bovine, Caenorhabditis Elegans, Cattle, Chicken, Hamster, Monkey, Pig; Suitable for WB, IP, IHC, IF, FC, ELISA; KD/KO validation (https://www.ptglab.com/products/TOM20-Antibody-11802-1-AP.htm#publications);

anti-Cleaved Caspase 3:Reactive with Human, Mouse, Rat, Monkey; Suitable for WB, IP, IHC, IF, FC; (https://www.cellsignal.com/products/primary-antibodies/cleaved-caspase-3-asp175-5a1e-rabbit-mab/9664);

anti-Ki67: Reactive with Bovine, Cat, Dog, Guinea pig, Hamster, Human, Mouse, Pig, Rabbit, Rat, Rhesus monkey, Sheep; Suitable for WB, IHC, IF, FC; Cell treatment validation (https://www.thermofisher.com/antibody/product/Ki-67-Antibody-clone-SP6-Recombinant-Monoclonal/MA5-14520);

anti-SOD2/MnSOD: Reactive with Human, Mouse, Rat; Suitable for WB;(https://www.abcam.com/sod2mnsod-antibody-ab13533.html); KO validation in manuscript;

anti-GNPAT: Reactive with Human, Mouse, Rat, Hamster; Suitable for WB, IP, IHC, IF, ELISA; KD/KO validation (https://www.ptglab.com/products/GNPAT-Antibody-14931-1-AP.htm)

anti-AGPS: Reactive with human; Suitable for WB, IF, IHC; (https://www.sigmaaldrich.com/US/en/product/sigma/hpa030211); KO validation in manuscript;

anti-FAR1: Reactive with Human, Mouse, Rat; Suitable for WB, ICC/IF, IHC, IHC-P; (https://www.novusbio.com/products/far1-antibody_nbp1-89847); KO validation in manuscript;

Total OXPHOS Human WB Antibody Cocktail: Reactive with Human; Suitable for WB; (https://www.abcam.com/total-oxphos-human-wb-antibody-cocktail-ab110411.html);

anti-ATP5A: Reactive with Mouse, Rat, Cow, Human, Drosophila melanogaster; Suitable for WB, IHC-P, ICC/IF, FC; (https://www.abcam.com/atp5a-antibody-15h4c4-mitochondrial-marker-ab14748.html);

anti-vinculin: Reactive with Ch, H, M, R, Rb; Suitable for IP, WB, IHC; (https://www.emdmillipore.com/US/en/product/Anti-Vinculin-Antibody-clone-V284,MM_NF-05-386);

aanti-pimonidazole rabbit antisera: Specifically reactive with compound pimonidazole; Suitable for IHC; (http://site.hypoxyprobe.com/knowledge-center-articles/HP3-Hypoxyprobe-Omni-Kit-Insert-2020.pdf)

Eukaryotic cell lines

Policy information about cell lines and Sex and Gender in Research

Cell line source(s)

Human PATC cell lines (PATC53, PATC66, PATC108, PATC118, PATC124, and PATC148) were generated from pancreatic duct adenocarcinoma by patient derived xenograft (PDX) models as previously described (Carugo et al., 2016). All PATC cell lines

	were cultured and treated with compounds at early passages (within 20 passages).
Authentication	PATC cells were authenticated by Short Tandem Repeat (STR) analysis.
Mycoplasma contamination	All cell lines were detected mycoplasma negative.
Commonly misidentified lines (See ICLAC register)	N/A

Animals and other research organisms

Policy information about studies involving animals; ARRIVE guidelines recommended for reporting animal research, and Sex and Gender in Research

Laboratory animals	Nude mice (Strain #002019) were purchased from The Jackson Laboratory. Both 6-8 weeks male and female mice with similar weight were used for all in vivo experiments.
Wild animals	No wild animals were used in this study.
Reporting on sex	This project is not a sex-biased study. Both Male and female mice were used.
Field-collected samples	This study didn't involve samples collected from field.
Ethics oversight	All mice were operated and treated under protocols approved by M.D. Anderson's Institutional Animal Care and Use Committee (IACUC).

Note that full information on the approval of the study protocol must also be provided in the manuscript.

Flow Cytometry

Plots

Confirm that:

- [x] The axis labels state the marker and fluorochrome used (e.g. CD4-FITC).
- [x] The axis scales are clearly visible. Include numbers along axes only for bottom left plot of group (a 'group' is an analysis of identical markers).
- [x] All plots are contour plots with outliers or pseudocolor plots.
- [x] A numerical value for number of cells or percentage (with statistics) is provided.

Methodology

Sample preparation	All PATC cell lines were cultured in standard incubation conditions (5% CO2, 37°C) and treated with compounds at early passages (within 20 passages).<br>ForCell death assay, 300000 cells per well were seeded in 6-well plates and treated with indicated concentrations of compounds for specific durations. Cells were harvested, stained with propidium iodide (PI, BD-Pharminogen#51-66211E), and detected within 1 hour (hr) of staining by using a Gallios flow cytometer (Beckman Coulter, Inc. Brea, CA) and following the manufacturer's instructions.<br>Mitochondrial reactive oxygen species (ROS) and lipid peroxidation detection. 3×105 PATC cells were plated and treated with indicated compounds for specified durations. Cells were harvested, washed with cold PBS, and stained with 5 µM MitoSOX (Life Technologies, #M36008) or 1 µM BODIPY 581/591 C11 (Thermo Fisher Scientific #D3861) for mitochondrial ROS detection or lipid ROS detection, respectively, for 20 min at 37 °C. Cells were then washed with cold PBS twice and filtered into single-cells suspensions. Cells stained for MitoSOX were then detected by a PE-Texas red filter and separated by flow cytometry. The PE-Texas Red filter was also used for detecting reduced BODIPY-C11, and FITC was used for detecting oxidized BODIPY-C11. Approximately 10000-20000 cells were collected and analyzed with Kaluza and Flowjo (Version 10.6.2) software. Peak shifts detected by MitoSOX-based flow cytometry indicated the medium fluorescence intensity (MFI) of mitochondrial ROS production. Further, the ratio oxidized/reduced BODIPY-C11 indicated levels of lipid peroxidation in each group.
Instrument	BCI Gallios Analyze
Software	Kaluza and Flowjo (Version10.6.2)
Cell population abundance	10000-20000 cells were collected for each treatment.
Gating strategy	Forward vs side scatter (FS-INT & SS-INT) was used to identify the suitable cells population and exclude the dead cells or debris. The FS/FS-INT were used to remove the doublets from total cell population. Target cells were gated out based on gating of unstained cells and single stained cells using appropriate channels.

- [x] Tick this box to confirm that a figure exemplifying the gating strategy is provided in the Supplementary Information.
