# Supplementary material for: Ether phospholipids are required for mitochondrial reactive oxygen species homeostasis
Source: Nat Commun. 2023 Apr 17;14:2194. doi: 10.1038/s41467-023-37924-9 (PMC10110566; doi:10.1038/s41467-023-37924-9)
Supplement: Supplementary file 4 — Description of Additional Supplementary Files [file 41467_2023_37924_MOESM4_ESM.pdf]

Description of Additional Supplementary Files

File name: Supplementary Data 1

Description: Global polar metabolomics in PDX lines. Comparation of basal level of 396 metabolites in 4 PDX lines, PATC66 / 108 / 124 / 148. Values were normalized in terms of raw area counts and BRADFORD protein concentration and rescaled to set the medium equal to 1.

File name: Supplementary Data 2

Description: Whole cell lipidomics in PDX lines. Comparation of basal level of over 1000 lipids in 4 PDX lines, PATC66 / 108 / 124 / 148. Values were normalized in terms of raw area counts and BRADFORD protein concentration and rescaled to set the medium equal to 1.

File name: Supplementary Data 3

Description: Lipidomics in purified mitochondria of PDX lines. The identified lipid molecules were quantified by normalization to a lipid class-specific internal standard. The amounts in pmoles of individual lipid molecules (species of subspecies) of a given lipid class were summed to yield the total amount of the lipid class.
